# Altitudinal, temporal and trophic partitioning of flower-visitors in Alpine communities

**DOI:** 10.1038/s41598-018-23210-y

**Published:** 2018-03-16

**Authors:** Vincent Lefebvre, Claire Villemant, Colin Fontaine, Christophe Daugeron

**Affiliations:** 1 Muséum national d’Histoire naturelle, ISYEB, UMR 7205 MNHN, CNRS, UPMC, EPHE, 45 rue Buffon, CP 50, 75005 Paris, France; 2 Muséum national d’Histoire naturelle, CESCO, UMR 7204 MNHN, CNRS, UPMC, 55 rue Buffon, 75005 Paris, France; 3 Muséum national d’histoire naturelle, Centre National de la Recherche Scientifique, Mécanismes adaptatifs et évolution, UMR 7179 MNHN-CNRS MECADEV, CP 50, 45 rue Buffon, 75005 Paris, France

## Abstract

The cross-pollination of most alpine plants depends on insects, whose altitudinal distribution is limited by temperature. However, although global warming is causing shifts in temporal and spatial species distribution, we are still largely unaware of how plant-pollinator interactions change with elevation and time along altitudinal gradients. This makes the detection of endangered interactions and species challenging. In this study, we aimed at providing such a reference, and tested if and how the major flower-visiting insect orders and families segregated by altitude, phenology and foraging preferences along an elevational gradient from 970 m to 2700 m in the Alps. Flies were the main potential pollinators from 1500 m, as bees and beetles decreased rapidly above that limit. Diptera, Coleoptera and Hymenoptera differed significantly in the angiosperm assemblages visited. Within Diptera, the predominant group, major families segregated by both phenology and foraging preferences along the gradient. Empidids, muscids and anthomyiids, whose role in pollination has never been investigated, dominated the upper part of the gradient. Our results thus suggest that flies and the peculiar plants they visit might be particularly at risk under global warming, and highlight the blatant lack of studies about critical components of these rich, yet fragile mountain ecosystems.

## Introduction

Mountains are biodiversity hotspots with high endemism that harbour one-quarter of terrestrial life, including a remarkably high plant richness in alpine life zones^1^. This proves especially true for angiosperms in the southern Alps^2^, whose specific diversity is now well documented^3^. Most alpine flower-plants depend on insects for pollination^4–8^, forming complex networks of dependencies among species^9^. Because climate change is expected to strongly affect mountain ecosystems^1^, it becomes urgent to develop a better knowledge of the insects involved in the pollination of alpine plants, as well as to investigate the factors structuring plant-pollinator networks along altitudinal gradients. Additionally, such gradients represent valuable baselines to detect species declines, range shifts and extinction risks, notably in the current climate change issue^10^.

In response to elevation, patterns of abundance and species richness often reveal a monotone decrease or a mid-elevation peak for both plants and animals, but vary among taxa and geographic areas^11–14^. Those patterns are partly shaped by abiotic factors physically tied to elevation^15^ that become life-limiting at high altitudes, including a temperature lapse rate of 6 °C per 1000 m on average^16^, a decline of available land area, and a decreasing atmospheric pressure^17^. Additional abiotic factors affecting living organisms such as precipitation, soil moisture, persistence of snow cover or seasonality often change with elevation, but also depend on local peculiarities^15,18^. By significantly reducing the duration of the growth period^19^ and metabolic rates^20^ in insects, harsher environmental conditions influence their survival and hence their altitudinal distribution range^13^. Because the different anthophilous insect groups seem affected to various degrees^21^, communities of pollinators involved in the reproduction of mountain angiosperms are expected to vary along elevational gradients.

However, studies focusing on altitudinal gradients of plants-pollinators interactions are scarce^7,22–26^ and often have significant sampling biases. Most of them focus on minimal gradients with only two different elevations, and lack a reproducible sampling protocol (Supplementary Table S1). In spite of their very different locations, durations and methods, they agree that both abundance and diversity of pollinators decrease with increasing elevation, and that the proportion of flower-visiting flies increases while the proportion of bees and beetles decreases. The predominance of flies in alpine flowers has been confirmed in several distant locations^27–34^ and this pattern was also observed at high latitudes^35–39^, which suggests that low temperatures favor the dominance of flies as flower-visitors and pollinators in cold habitats.

Besides elevation, two major dimensions of anthophilous insects’ ecological niche contribute to shape the structure of plant-pollinator networks along an altitudinal gradient. First, phenology can vary among flower-visiting groups at a given elevation, but also among elevations for a given group. From a plant species viewpoint, it means that the assemblages of potential pollinators may vary in both time and space over the gradient and favorable season. To our knowledge, this issue has never been investigated in temperate mountains, although phenology was found to be the main driver of species’ interactions in a bird–flowering plant network in the high Andes of Peru^40^ and is known to shape pollination networks in lowland areas^41^. Second, insect species involved in pollination interactions can exhibit different foraging preferences, resulting in different dominant pollinator group among different flowering plant species. To what extent the sets of flowers visited by the different orders or families of anthophilous insects overlap across elevation gradients is still not widely known.

In the present study, we examined how the abundance and species richness of flower visitors varied along an altitudinal gradient, in order to understand how they segregate according to space (elevation), time (phenology) and trophic resource (species of plants visited). We especially investigated (1) how elevation, time and taxa influenced the abundance and richness of foraging insects, at both order and family levels; (2) whether the species belonging to those taxa overlapped in their choice of angiosperm species visited, at each elevation level and across the entire gradient. We suggest and discuss several hypotheses involving ecological and historical factors that may explain the patterns observed, as well as their implications in a context of global climate change.

## Results

### Species identification and community composition

At the 13 study sites, 5502 flower-visiting insects were collected or identified in field, representing a minimum of 521 species and 206 genera in 69 different families (Supplementary Table S16). 4224 of them were identified to species level, 583 to morpho-species, 387 to groups of species, 287 to family and 21 to order level. They were caught foraging on 121 different flowering-plant species belonging to 86 genera in 31 families (Supplementary Table S15). More than 99.8% of them belonged to the four major orders of anthophilous insects: Diptera accounted for 49.2% (N = 2706, 253 species) of the interactions, Hymenoptera 27.5% (N = 1511, 134 species), Coleoptera 18% (N = 989, 102 species) and Lepidoptera 5.2% (N = 289, 29 species).

### Effects of time and altitude on the abundance and species richness of flowering plants

After model simplification, we found that the interaction between Julian day (JD) and the quadratic term for elevation significantly influenced both abundance and species richness of flowering plants along transects (Supplementary Table S4 and S5). Angiosperm diversity increased with altitude and saturated around 2000 m. The phenological peaks of abundance and richness varied with altitude, with a delayed peak at higher elevations (Supplementary Fig. S1).

### Altitudinal and temporal variations in abundance and diversity of anthophilous insect orders and overlap in their resource use

Both the abundance and species richness of flower visitors were significantly influenced by the interaction between insect order and the quadratic term for the date (JD²), and by the interaction between order and altitude or its quadratic term. This indicates that the main orders have different phenologies, and that elevation affected the abundance and species richness of anthophilous insects in a direction and with an intensity that depended on their taxonomic order.

**Figure 1:**
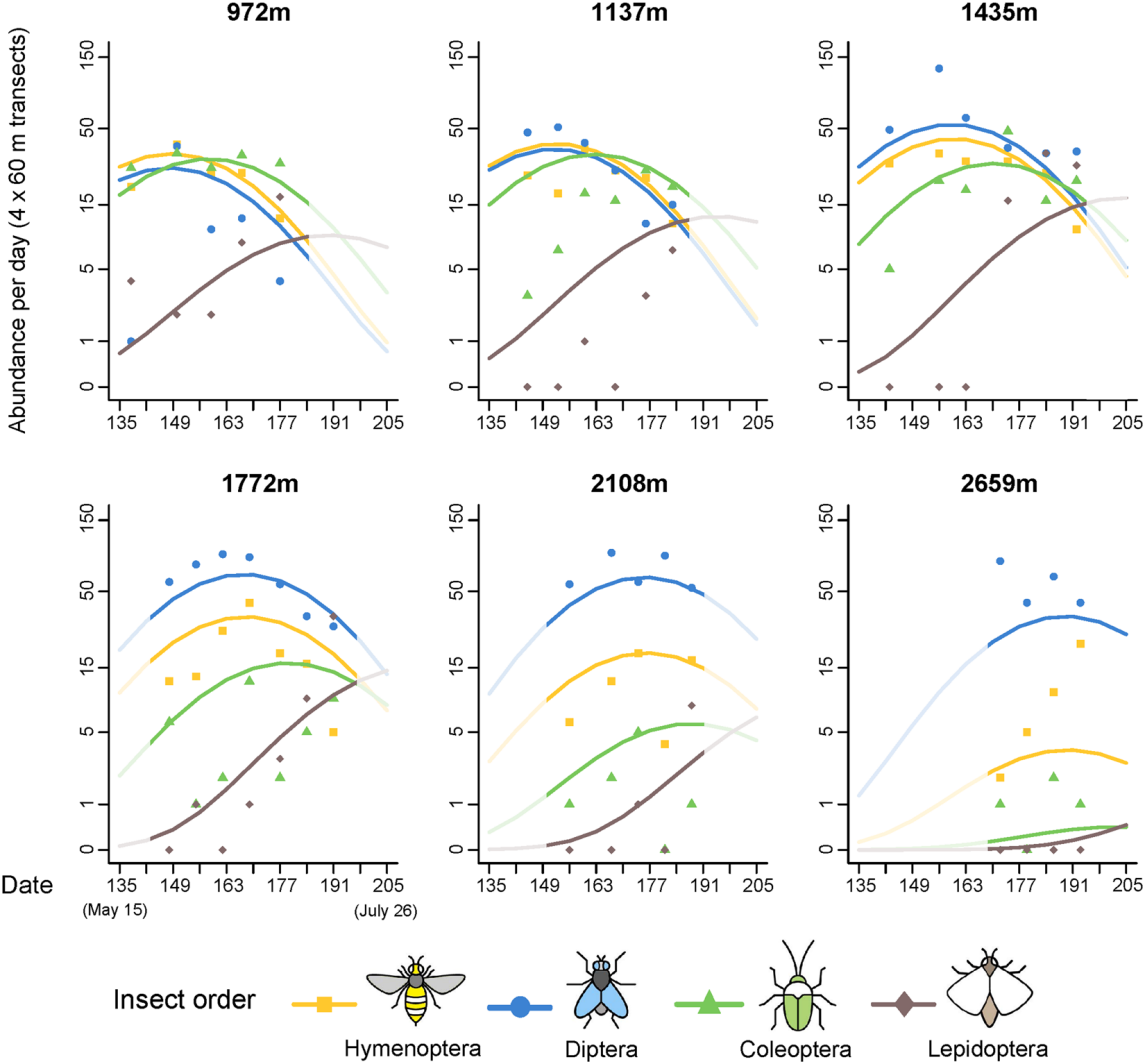
Abundance of the four major anthophilous orders over time at six different elevations along the gradient. Lines represent the predictions of the most parsimonious model and dots the observed abundances (associated graphs for species richness in Supplementary, Fig. S3). Insect icons created by Iconicbestiary @ Freepik.com and modified by the authors (V.L.).

**Figure 2:**
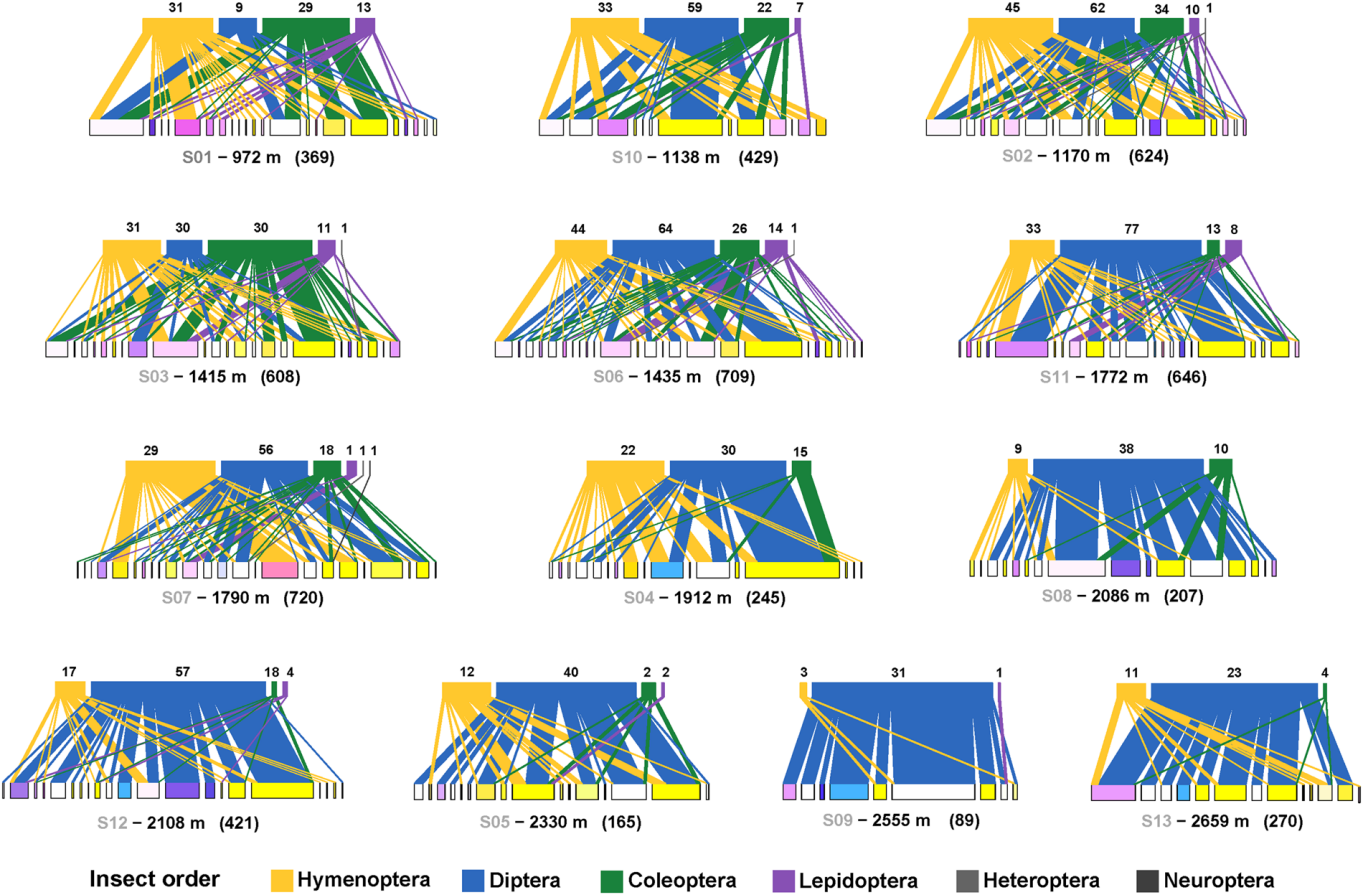
Flower-visitation networks for each study site, showing the changes in the proportion of each major anthophilous order (upper level boxes) with elevation. Each box of the lower level represents a species of flowering plant and has the corresponding flower colour. The number indicated above each order bar is the number of insect species collected in that site and order. Site code, elevation and total number of visits are given below each network.

Model predictions indicated strong differences in response to altitude among insect orders. While both visitation frequency and species richness of Hymenoptera, Coleoptera and Lepidoptera decreased rapidly with elevation above 1400 m, Diptera increased in species richness and abundance to reach a peak around 2100 m. (Fig. 1 and Supplementary Fig. S3). At approximately 1500 m elevation, the major flower-visiting order shifted from either Hymenoptera, Diptera or Coleoptera, depending on the site (and whether or not honey bee numbers are taken into account, see Supplementary Fig. S2), to Diptera at every site (Fig. 2).

The insect orders also exhibited differences in phenology. The abundance of Hymenoptera and Diptera peaked simultaneously at each elevation, ten days before Coleoptera (Fig. 1). Regarding species richness, the temporal segregation of insect orders was slightly more pronounced: flies reached their maximum one week before bees and two weeks before beetles (Supplementary Fig. S3). Butterflies exhibited a distinct temporal pattern with no abundance or diversity peaks at any elevation. Abundances and species richness peaks of flies, bees and beetles were delayed by one week on average for every additional 300 m increase in altitude (Fig. 1 and Supplementary Fig. S3).

**Figure 3:**
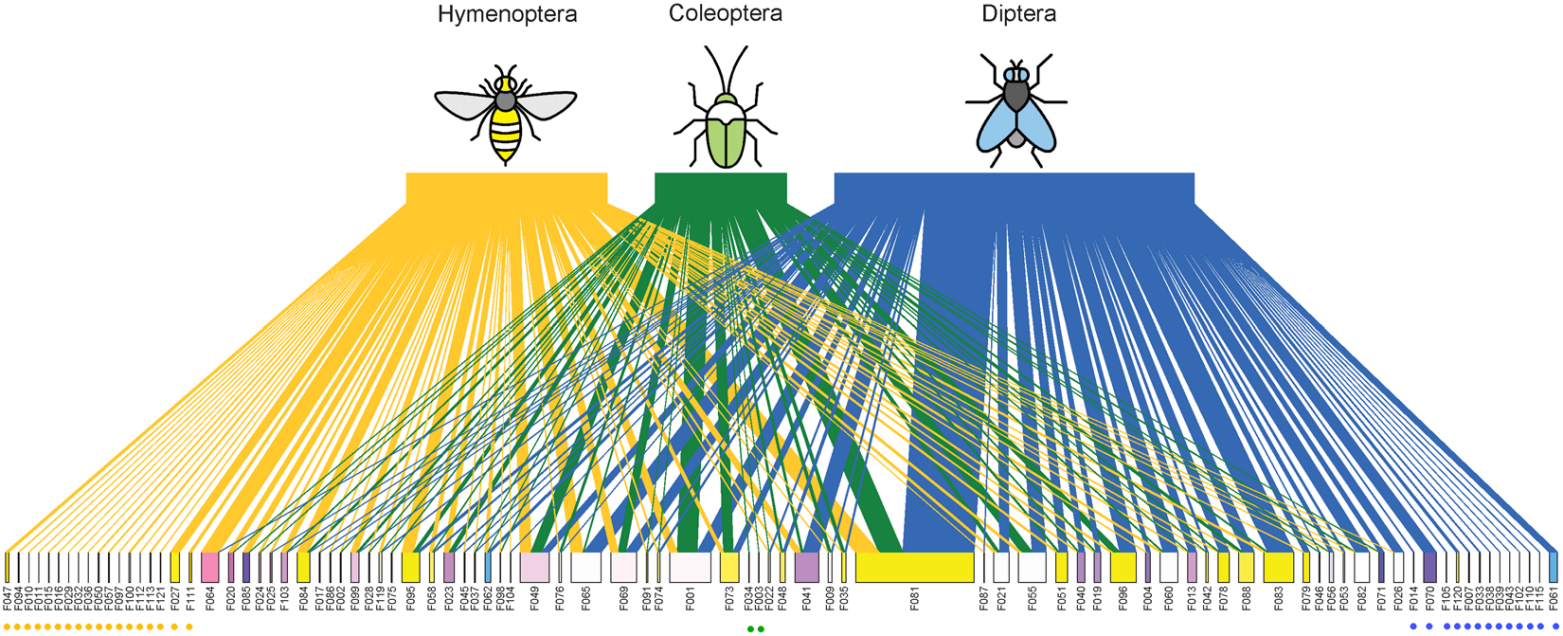
Plant-visitor network illustrating the choice of plant species visited by Hymenoptera (yellow), Coleoptera (green), and Diptera (blue). Plants visited only by species of a single order are indicated by a dot with the colour of that order. For legibility reasons, the species-level networks corresponding to the overlap analyses between each pair of orders are provided in Supplementary, Fig S11. Flower codes are listed in Supplementary, Table S15. Insect icons created by Iconicbestiary @ Freepik.com and modified by the authors (V.L.).

Our analysis of the visitation networks revealed that the resource overlap among species from different insect orders was lower than expected. The pairwise Bray-Curtis dissimilarities between Diptera, Hymenoptera and Coleoptera species calculated per site were most often higher than random expectation, but rarely significantly. However, this tendency was highly significant when considering all the sites (Jost’s combined significance test, p < 0.0001; Table S7). This result indicates that across the elevation gradient, the species from different orders exhibited different foraging preferences: among the 90 plants visited by these 3 orders, 20%, 14% and 2% of them were exclusively visited by bees, flies and beetles respectively (Fig. 3). Lepidoptera were excluded from the analysis due to their insufficient number of visits per site.

### Altitudinal and temporal variations in abundance and diversity of the main anthophilous families of Diptera and overlap in their resource use

The abundance and species richness of flower-visiting flies were significantly influenced by two-ways interactions between date and altitude, fly family and altitude and fly family and date (Supplementary Table S8). This indicates that both the phenology and the response to increasing elevation varied among dipteran families.

**Figure 4:**
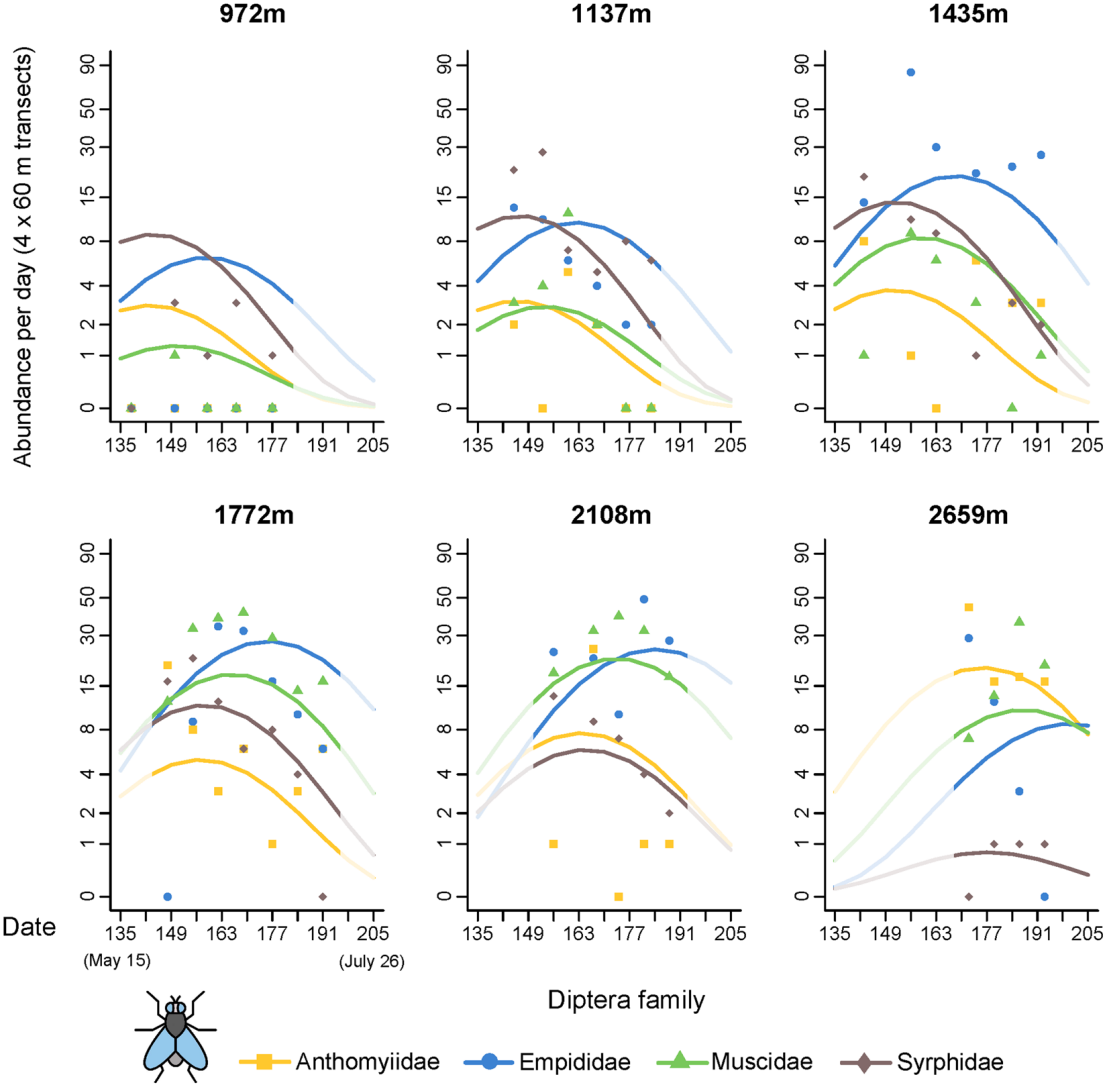
Abundance of the four major dipteran families over time at six different elevations along the gradient. Lines represent the predictions of the most parsimonious model and dots the observed abundances (associated graphs for species richness in Supplementary, Fig. S4). Insect icon created by Iconicbestiary @ Freepik.com and modified by the authors (V.L.).

The model predictions showed that the main anthophilous fly families reached their highest visitation frequency at different altitudes, with syrphid abundance peaking at mid-elevation around 1500 m, empidids around 1800 m, and anthomyiids and muscids above the tree line, from 2300 m. As a result, the predominant fly family within the anthophilous Diptera community varied along the altitudinal gradient, with syrphids being the main visitors at the lower part of the gradient, empidids at the intermediate part and anthomyiids and muscids at the highest elevations (Fig. 4).

Regarding the species richness, syrphids with 93 species were by far the most speciose family among flies, but also in the whole study. They exhibited a higher diversity than any other fly family from the lower limit of the gradient up to 1900 m. Empidids were the second most speciose family with 45 species, their maximum species richness occurring at intermediate altitudes between 1800 and 2100 m. Anthomyiids (25 species) and muscids (33 species) showed a maximum species richness between 2200 and 2500 m, where they were the most speciose families (Supplementary Fig. S4).

The phenology of fly families also varied. When analyzing visitation frequencies, for any given altitude, syrphids and anthomyiids had their abundance peaks eight days before muscids, which peaked themselves ten days before empidids. Abundance peaks of the four main fly families were also delayed with increasing elevations (Fig. 4).

**Figure 5:**
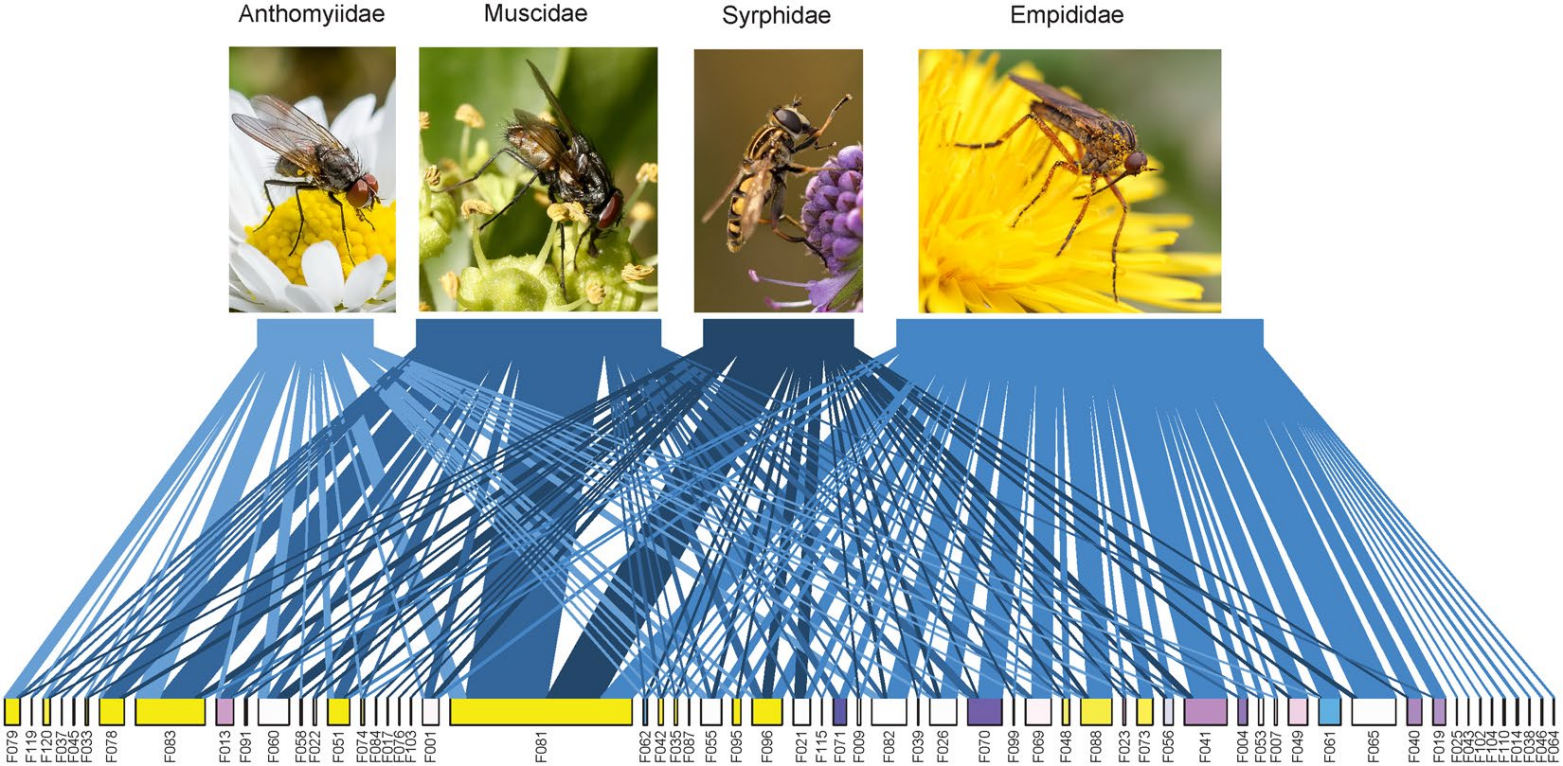
Plant-visitor network illustrating the choice of plant species visited by the four main Diptera families (from left to right: Anthomyiidae, Muscidae, Syrphidae, Empididae). For legibility reasons, the species-level networks corresponding to the overlap analyses between each pair of fly families are provided in the Supplementary, Fig S12. Flower codes are listed in Supplementary, Table S15. Photos: Anthomyiidae, Syrphidae, Empididae: PixaBay.com; Muscidae: Christophe Lauriaut.

The diet overlap among species of the major fly families was lower than expected. When comparing the plant species visited, pairwise distances between each couple of fly families (Anthomyiidae, Empididae, Muscidae and Syrphidae) were all significantly larger than expected at random, with the exception of the Syrphidae-Anthomyiidae pair (Supplementary Table S9). It indicates that over the altitudinal ranges where several of those fly families were well represented, they did not interact with the same assemblages of flowering-plants. Among the 61 angiosperms they visited, empidids were the exclusive fly-visitors for 9 (15%) species and syrphids for 4 (6.5%) species (Fig. 5).

### Altitudinal and temporal variations in abundance and diversity of the main anthophilous families of Hymenoptera and overlap in their resource use

The flower-visit frequency and species richness of Hymenoptera were significantly influenced by the two-ways interactions between altitude and both date and family. This means that the effect of elevation on both the abundance and richness of hymenopterans varied in time, but also among families. However, their abundance but not their species richness was influenced by the interaction between family and date, showing that all families reached their maximum species richness at the same time, but not their maximum abundance (Supplementary Table S10).

**Figure 6:**
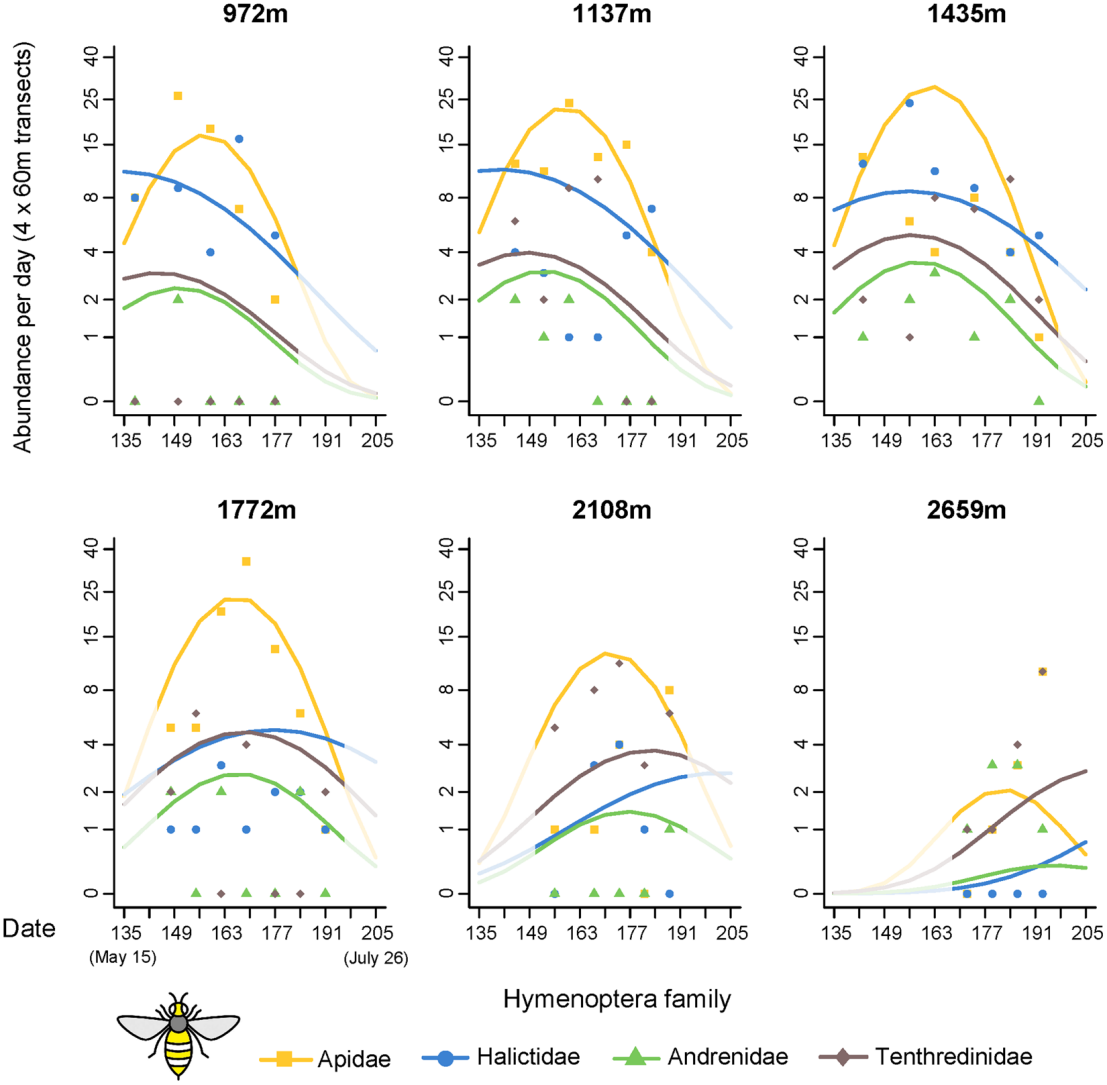
Abundances of the four major hymenopteran families over time at six different elevations along the gradient. Lines represent the predictions of the most parsimonious model and dots the observed abundances (associated graphs for species richness in Supplementary, Fig. S5). Insect icon created by Iconicbestiary @ Freepik.com and modified by the authors (V.L.).

Model predictions show that above 1500 m, three of the four major families (Andrenidae, Apidae and Halictidae) had decreasing visit frequencies and species richness with increasing elevation. Sawflies (Symphyta, Tenthredinidae), however, revealed a different pattern with their abundance almost not affected by elevation, making them the most abundant hymenopteran group in the upper part of the gradient (Fig. 6). Halictidae, whose number of visits was lower than that of Apidae, was the most speciose bee family (38 species) with the maximum species diversity among Hymenoptera up to 2000 m elevation; conversely, Apidae, the most abundant family, ranked second in bee species richness with 26 species, including 18 species of bumblebees (Fig. 6 and Supplementary Fig. S5). The honey bee, which accounted for more than 38% of all Hymenoptera visits, was responsible for this Apidae prevalence: removing it from the analysis resulted in Halictidae being both the most abundant and speciose group from the lower limit of the gradient up to 1800 m. (Supplementary Fig. S5 and S6).

### Altitudinal and temporal variations in abundance and diversity of coleopteran flower-visitors

The abundance and species richness of Coleoptera were both significantly influenced by the three-way interaction between family, JD and altitude (Supplementary Table S12). This indicated that the phenology differed among beetle families and elevations, with effect of elevation depending on families.

Model predictions showed that both the abundance and richness of anthophilous beetles decreased with increasing elevation and especially above 2000 m, with the exception of Rutelidae and Cerambycidae peaking at mid-elevation around 1400 m (Supplementary Figs S7 and S8). Flower-visiting beetles disappeared completely above 2300 m altitude, except for some leaf beetles in the genus *Cryptocephalus* (Chrysomelidae) and small anthophilous species of Staphylinidae. Regardless of insect order, long-horned beetles (Cerambycidae) and leaf beetles were among the most abundant families in the lower part of the gradient up to 1500 m, both including 16 different species.

The major beetle families exhibited very distinct phenologies over their elevation range. Long-horned beetles’ peaks of abundance and richness were almost not delayed with increasing elevation, and occurred almost one month after those of leaf beetles over the lower part of the gradient. However, as the phenology of leaf beetles showed an average delay of 10 days for every 300 m altitude increase, these two beetles families exhibited a simultaneous maximum abundance and richness around 1600 m altitude (Supplementary Figs S7 and S8).

## Discussion

So far as we are aware, this is the first study investigating plant-visitor interactions along an elevation gradient with such an extensive altitudinal range, a fine altitudinal and taxonomic resolution, and a sustained sampling effort including all flower-visiting insects during peak flowering at all elevations. Our findings highlight the differences in response to altitude among various natural groups of insects including 4 orders and 17 families, and the resulting changes in flower-visitor assemblages along the gradient. At order-level, a major shift occurs around 1500 m, altitude above which flies constantly predominate as flower-visitors, and below which the most conspicuous flower-visiting order varied among sites with no consistent altitudinal pattern. This transition is due to the peculiar elevational pattern of abundance and richness exhibited by anthophilous flies over most of the gradient, which is unique within insects at this taxonomic level^42,43^. Conversely bees, beetles and butterflies conformed to a pattern commonly observed in arthropods with either a mid-elevation peak or a monotonical decrease in abundance and species diversity over the entire gradient^44–46^. Flies therefore represent the most diverse and abundant potential biotic vector for the diurnal cross-pollination of alpine angiosperms.

Elevation also shaped the assemblages of anthophilous insects at family level at least within the two main orders. Altitudinal structuring was especially marked among fly families, with each main family adapted to a different elevation range. Hoverfly abundance and richness peaked around 1500 m and became scarce above 2000 m, as for most insects^11^. Such scarcity of syrphids at high altitude has already been found^47^ and echoes what is found at high latitudes^48–52^, suggesting that they only play a minor role in the pollination of alpine flowers. On the contrary, empidids, muscids and anthomyiids all peaked between 1800 and 2500 m; a similar pattern has recently been found for empidids along an altitudinal gradient in the tropics^53^. Unlike hoverflies, the importance of those families for pollination has been poorly addressed. Therefore, further studies are needed to investigate to what extent the reproductive success of alpine plants benefits from their visits. Within Hymenoptera, the decreasing abundance and richness with altitude of bees – congruent with the results of Hoiss and colleagues^54^ – contrasts with the altitudinal response of sawflies. This questions the existence of a phylogenetic effect. Bee families are closely related to each other while sawflies are distant relatives to bees, with both groups having very different life cycles and larval life patterns: free-living sawfly larvae feed on plant leaves, while bee larvae are hidden in a nest and consume the pollen harvested by their mother. Finally, no clear altitudinal structuring was found among butterfly families, mostly because their abundance and diversity became too low - or they were absent - over the upper half of the gradient. The different anthophilous insect groups present at high elevations are not closely related to each other (*Bombus* and Symphyta within Hymenoptera; Anthomyiidae, Empididae and Muscidae within Diptera), showing that life-history traits related to cold tolerance have emerged independently in several lineages within insects.

Our results are in agreement with the scant literature on flower-visitor networks at high altitude but also at high latitudes^35–38^, suggesting that the limiting abiotic factors structuring flower-visiting communities are mainly temperature and humidity. Although exceptions exist, flies prefer cool and moist conditions^29,53^, while bees search for warmer and drier habitats^55^. Life-history traits that could favour flies over other anthophilous groups in alpine areas still need to be investigated. However, larval stages, which most often last much longer than adult stages in insects, probably focus most of the selective pressures that limit their spatial distribution. The majority of anthophilous beetles, whose larval growth depends on fresh, dead or decayed wood simply cannot thrive above the timberline. The scarcity of solitary bees at altitude could result from various foraging or nesting constraints, while in contrast, the success of bumblebees is likely favoured by both their rapid development allowed by sociality^56^, and their ability to fly and forage at lower temperatures than small bees^29^.

Several families of flies appear particularly well adapted to mountain habitats (i.e. empidids, muscids, anthomyiids), and, although we do not know the biology of most species thriving at altitude, the few documented cases suggest that their larvae develop in moist to wet soils or in dung, where they feed on small organisms^57,58^. Sheep and cattle grazing, cool temperatures and snowmelt contribute to the abundance of those suitable habitats at mid and high elevations. Additional factors, including the large amount of time they spend basking in sun-exposed flowers to thermoregulate^59–62^, also promote the conspicuousness of Diptera as flower-visitors at high elevations. This Diptera-specific behaviour implies that high-altitude, flies-dominated pollination networks are much more sensitive to global warming than lowland, bees-dominated networks: by making fly visits related to thermoregulation unnecessary, rising temperatures are expected to quickly and profoundly disrupt the structure of high-altitude plant-pollinator networks.

As anticipated, both flowers and insects peaked later at higher elevations, where snowmelt occurs later and the favourable season is shorter. No marked temporal segregation appeared between the three major orders of anthophilous species. Diptera and Hymenoptera had a similar phenology at each elevation, and, although Coleoptera peaked slightly later, the three orders had a simultaneous period of high abundance over the lower part of the gradient. Those overlapping phenologies are probably due to a synchronicity with the flowering peak of angiosperms. However, as for the vertical partitioning, the main anthophilous fly families exhibited different temporal patterns. All along the gradient syrphids and anthomyiids peaked slightly before, muscids during, and empidids just after the peak of flowering. This pattern results in the high abundance of interactions between syrphids and early-blooming plant species (i.e. *Ranunculus* spp., *Meum athamanticum*), and between empidids and late-blooming plant species (i.e. *Phyteuma* spp., *Knautia* spp., *Scorzonera* spp., *Persicaria bistorta*). Consequently, over the altitudinal ranges where these families co-occur, they complement each other in time for the flower visitation and the potential pollination of the angiosperm community.

For the first time, we show that the three main orders, as well as all but one pair of fly families, segregate significantly by their choice of flowering-plant species along the gradient. As a consequence, several plant species were visited by a unique order. The mechanisms that led the anthophilous insect groups to exploit different floral resources were not tested in this study, but could be partly related to floral traits resulting from flower-insect coevolution. For instance, zygomorphic flowers were visited mostly by bees, which is consistent with the association between bilateral floral symmetry and bee pollination reported in the literature^63^. This trophic partitioning among Coleoptera, Diptera and Hymenoptera suggests that at low and mid- elevations, the three orders could pollinate different assemblages of flowering-plant species. Similarly, the differences in foraging patterns among flies indicate that when several families are present over a given altitudinal range, they might provide pollination to different guilds of angiosperms. Moreover, the fact that the flowers visited by flies differ significantly among families and from those visited by the other orders challenges their alleged generalism as flower visitors and pollinators, which should encourage further studies in this area.

The networks analyzed in this study consist of flower visits by insects, whose associated pollination effectiveness can vary depending on the amount of pollen deposited by the insect on the flower’s stigma. Although these visitation networks therefore cannot be directly interpreted as pollination networks, there is an indirect evidence that some of the visitors are pollinators: of the 121 plants species in the study, 95% (115) require insects for cross-pollination and 71% (86) are totally dependent on insects for reproduction (Supplementary, Table S15). As a consequence, the mere fact that these angiosperm species survive and thrive proves that anthophilous insects do pollinate them successfully. Additional research such as single-visit experiments would be required to properly assess the pollination effectiveness of insects of each visited flower, but this was out of the scope of our study. Along the elevation gradient, the reliance of the vast majority of plants on pollinating insects for reproduction implies that potential changes in the flower visitation patterns are expected to be traduced in changes in reproductive performance for those plant species.

Several considerations that could influence our results have to be taken into account. First, sampling periods stopped at latest at the end of July, probably explaining the strikingly low abundance of Lepidoptera, which seemed to have a later phenology compared to other orders. The fact that sampling took place only during the day could also introduce a bias, given that most lepidopterans are nocturnal^64^ and were thus not included in this study. Second, the relative abundance and species richness of each group over the gradient, as well as the transition altitude above which Diptera become predominant, are likely to vary with the geographic area, but also with local peculiarities, year and climatic conditions. For instance, this transition occurs around 700 m altitude in Tasmania^65^ whereas we observed it around 1500 m; it is very likely that the higher the latitude, the lower this transition altitude. Third, although pollination occurs at species level and the abundance of visits was very unevenly distributed among anthophilous species within each group, we performed our analyses at order and family levels and thus do not take these ratios into account. For instance, only 5.2% of the flower-visiting species were responsible for more than 50% of the 5500 interactions, while 39% were involved in only one interaction. Therefore, additional studies at a finer taxonomic scale are necessary to understand the role of each species in pollination networks.

Elevational gradients allow the investigation of plant-visitor networks in a changing environment where temperature depends on altitude, and where niche partitioning among insect pollinator communities can occur mainly through spatial, temporal and trophic segregation. Our results show that the different orders segregated by both altitude and resource, and that within flies, the predominant order, families segregated by the three niche dimensions along the gradient. Further, they point to several key-taxa on which future investigations should focus: although Anthomyiidae, Empididae and Muscidae are predominant over a great part of the gradient, and therefore the main available visitors of alpine flowers, their foraging behaviour and pollination efficiency have never been formally studied. Moreover, while the abundance of muscids has been reported as the main predicator for seed set in *Dryas integrifolia*, a key plant-species in the arctic, a first study reported their decline over the last years in the same area^66^.

In a context where global warming is expected to change the spatio-temporal distribution of anthophilous insect, our results should provide valuable insights for conservation biologists investigating how plant-pollinator interactions will be affected. Bees are expected to shift toward higher elevation, whereas fly groups already present at the highest altitude levels can either disappear, shift their phenology or change their behavior, including their relationships with flowers. Whether plant-pollinator networks in alpine areas will face an impoverished or changed pollinator fauna depends on how the different anthophilous groups will respond to rising temperatures. In this context, further studies addressing the effects of warming at species level are needed, and would advance our ability to predict how global warming could reshape plant-pollinator networks in fragile alpine ecosystems.

## Material and Methods

### Study sites

The study was carried out in the Ubaye valley, near and in the Mercantour National Park, Southern Alps, France. The minimum and maximum elevations in the valley are 800 m and 2700 m respectively, with the highest peak reaching 3412 m. The 13 study sites consisted of montane, subalpine and alpine meadows at elevations ranging from 972 m to 2659 m altitude (Supplementary Table S2). We selected them for their distribution along the elevational gradient, their abundant flower cover, their low levels of anthropogenic disturbances and their type of management: late-summer mowing or grazing.

### Sampling protocol

Sampling sessions took place from mid-May to late July in 2014 (sites S01 to S09) and from late May to mid-July in 2015 (sites S10 to S13). At each site, sampling began shortly after snowmelt, when the first angiosperms began to bloom, and lasted until the end of the flowering peak or the meadow was mown or grazed. All the sampling sessions were conducted by the same observer (V.L.), only on non-rainy days and with at most a light wind. The protocol was inspired by Forup and colleagues^67^. At the beginning of both fieldwork seasons, a square plot of 60 × 60 m was delimited in each site. A sampling day at one site consisted of four 60 m-long transects connecting two opposite sides of the square plot, two in the morning (9:00–12:30) and two in the afternoon (13:00–17:00). The minimum duration of a transect without handling time was 15 min (no insect collected), and it was on average 35 min including handling time for the collection of flower-visiting insects.

Each transect was randomly established by selecting one of the plot edges from which the collector can face the sunlight and progress along the transect without his shadow scaring insects ahead. A point was drawn on this edge from which the transect started, with an end at the opposite side. Every insect visiting a flower and contacting its stamens or stigma, in a 1.5 m wide swathe on each side of the transect line, was either collected or identified by sight when capture wasn’t necessary (e.g. honey bees and several species of butterflies and beetles). Insects were collected using an aspirator or a killing jar with potassium cyanide. Sweep-net was not used to avoid disturbing other insects foraging along the transect. Specimens were stored in 96% ethanol together with related collection data: site, flowering plant species, time and date. A second pass was made to record the identity and abundance of every entomophilous plant species blooming along the transect.

### Processing of collected specimens

Collected insects belonging to the main families of Coleoptera, Diptera and Hymenoptera were identified to species or morphospecies by specialist taxonomists, and we identified the remaining specimens to family, genus or species level (Supplementary, Table S3). During the identification process, the location data have been lost for 113 bee specimens of the genus *Bombus* (representing 2% of the collected specimen and 6 species), which were therefore not included in the analyses. All the specimens included in the analyses are stored in the entomological collections of the Muséum national d’Histoire naturelle, Paris (MNHN).

### Statistical analyses

We performed all statistical analyses with R Software version 3.4.2 (https://www.R-project.org). Plant—flower-visitor networks were generated with the bipartite R package^68^. For all the analyses described below, we used generalized linear models (GLM) with Poisson residuals (or quasi-Poisson to account for overdispersion when necessary). We performed a stepwise model simplification procedure to identify the most parsimonious models. At each step we dropped the least insignificant variable or interaction based on p-value, and we compared the models using a Chi-square test to check whether reduction in the residual sum of squares of the simplified model was statistically significant. For each model, the normality of the residuals and homoscedasticity of variance were checked visually.

### Abundance and diversity of flowering plants

To investigate how the abundance and richness of plant communities varied with elevation and time, the dependent variables were either plant abundance or plant species richness, and the explanatory variables were elevation, Julian day (JD), as well as their quadratic terms (elevation², JD²) to test for curvilinear responses. We also included the interactions between elevation and JD, elevation and JD², and between elevation² and JD to test for temporal shifts in phenology peaks with altitude.

### Abundance and diversity of flower-visitors

Order-level analyses include every species of Diptera, Hymenoptera, Coleoptera and Lepidoptera, which together represented more than 99% of all recorded insect-flower interactions. Within each order, family-level analyses include the families exhibiting the highest abundances so that their cumulated number of visits represented at least 75% of the visits of the focus order: Andrenidae, Apidae, Halictidae, Tenthredinidae (Hymenoptera); Anthomyiidae, Empididae, Muscidae, Syrphidae (Diptera); Cerambycidae, Cetoniidae, Chrysomelidae, Oedemeridae, Rutelidae (Coleoptera); Adelidae, Hesperiidae, Nymphalidae, Zygaenidae (Lepidoptera).

We investigated how the abundance and species richness of those anthophilous groups varied with elevation, time and taxonomic group by fitting generalized linear models with either abundance or species richness as a dependent variable and elevation, JD, their quadratic terms, and taxonomic group as explanatory variables. We also included the following two-way interactions: elevation:JD, elevation:JD², elevation:taxonomic group, elevation²:JD, elevation²:taxonomic group, taxonomic group:JD, taxonomic group:JD², and the following three-way interactions: taxonomic group:JD:elevation, taxonomic group:JD²:elevation and taxonomic group:JD:elevation². For Lepidoptera, data were too scarce for family-level analysis of abundances and species richness, but corresponding tables and graphs are provided in Supplementary material (Tables S13 and S14; Figs S9 and S10).

### Overlap in floral resource use by two groups of insects

For each site, we tested whether the species belonging to two insect taxa T1 and T2 (two orders or families) differed significantly in their choice of plant species visited using a 3-steps randomization test. First, we calculated the average Bray-Curtis dissimilarity among all possible species pairs of the two taxa, D_obs_ (vegdist function of the “vegan” R package^69^). D_obs_ is bound between 0 and 1, where 0 means that all species pairs of the two taxa visit the exact same assemblage of flowering plants with the same frequency and 1 means they visit non-overlapping sets of plants. Second, we generated a null expectation D_0_ of this mean dissimilarity given the observed network structure. To do so, we shuffled the identity of species and re-calculated the average dissimilarity between all the species pairs of the two taxa T1 and T2 as in step 1. We repeated this operation 1000 times to obtain a null distribution of D_0_. Third, we compare D_obs_ to the distribution of D_0_. If D_obs_ is greater than 97,5% of the D_0_ values, then D_obs_ is significantly greater than expected under the null hypothesis, meaning that species belonging to T1 and T2 exhibit different foraging preferences at the focal site. Finally, to test for an overall pattern across the entire altitudinal gradient, we used the Jost’s formula for combining significance levels from multiple independent analyses^70^ on the p values obtained at all sites. Since each study site was several kilometers apart from others, we consider the different sites and related analyses to be independent. The formula is a generalization of Fisher’s combined probability test and is calculated as ${P}_{T1T2}=K\,\ast \,{\sum }_{i=0}^{N-1}\frac{{(-\mathrm{ln}(K))}^{i}}{i!}$, where N is the number of analyses and K is the product of the N corresponding p-values. To avoid analyzing under-sampled insect taxa, we only analyzed sites where both taxa had at least 50 visits and 10 species at order level, or 20 visits and 5 species at family level. As a consequence, this analysis was possible at order level but only within Diptera for family level.

## Electronic supplementary material

Supplementary material
