# Supplementary material for: Altitudinal, temporal and trophic partitioning of flower-visitors in Alpine communities
Source: Sci Rep. 2018 Mar 16;8:4706. doi: 10.1038/s41598-018-23210-y (PMC5856740; doi:10.1038/s41598-018-23210-y)

Altitudinal, temporal and trophic partitioning of flower-visitors in Alpine communities

Supplementary material

Vincent Lefebvre[1*], Claire Villemant[1], Colin Fontaine[2] and Christophe Daugeron[3]

[1] Muséum national d'Histoire naturelle, ISYEB, UMR 7205 MNHN, CNRS, UPMC, EPHE, 45 rue Buffon, CP 50, 75005 Paris, France

[2] Muséum national d'Histoire naturelle, CESCO, UMR 7204 MNHN, CNRS, UPMC, 55 rue Buffon, 75005 Paris, France

[3] Muséum national d'histoire naturelle, Centre National de la Recherche Scientifique, Mécanismes adaptatifs et évolution, UMR 7179 MNHN-CNRS MECADEV, CP 50, 45 rue Buffon, 75005 Paris, France.

* Corresponding author: vincent.lefebvre@mnhn.fr (ORCID: 0000-0002-6829-1938)

Table S1: Main altitudinal gradients of plants-pollinators interactions published.

Publication	Location	Reproducible protocol	Grain / limits of the gradient	Time
Müller 1880	European Alps	No	« Lowlands », « Alps », «Above the boundary of trees »	NC (years)
Heithaus 1974	Costa Rica	No	2 elevations: "lowland" and 3335m	1 year
Moldenke 1976	USA - California	No	0 to 4200 m but study sites spread all over California	8 years
Arroyo et al. 1982	Chile (centre)	Yes (beetles excluded)	3 ranges: 2200-2600 m, 2700-3100 m, and 3200-3600 m	6 months
Warren et al. 1988	USA - Utah	No	2 elevations: 1495 m and 3170 m (with 200 km in between)	3 weeks
Medan et al. 2002	Argentina (Andes)	No	2 elevations: 1900 m and 3300 m	6 days

Table S2: Altitude, GPS coordinates and sampling effort for each study site.

Code	Year	Altitude (m)	Latitude	Longitude	Transects (days)
S01	2014	972	44,399090	6,475160	20 (5)
S02	2014	1170	44,365687	6,629452	28 (7)
S03	2014	1415	44,355892	6,616068	24 (6)
S04	2014	1912	44,314721	6,750020	16 (4)
S05	2014	2330	44,259799	6.746169	16 (4)
S06	2014	1435	44,416401	6,767820	24 (6)
S07	2014	1790	44,398159	6,777288	20 (5)
S08	2014	2086	44,359412	6,787121	16 (4)
S09	2014	2555	44,344069	6,796272	16 (4)
S10	2015	1138	44,384271	6,592084	24 (6)
S11	2015	1772	44,399850	6,776540	28 (7)
S12	2015	2108	44,358870	6,786930	24 (6)
S13	2015	2659	44,335194	6,812772	16 (4)

Table S3: Taxonomists who identified the collected specimens, by taxonomic group.

Order	Taxon	Determinators
**Diptera**	Conopidae, Stratiomyidae, Syrphidae	Axel Ssymank
	Empididae	Christophe Daugeron
	Calliphoridae	Knut Rognes
	Tachinidae	Hans-Peter Tschorsnig
	Muscidae, partly Anthomyiidae	Adrian Pont
	Other families (above species level IDs)	Emmanuel Delfosse, Christophe Daugeron, Vincent Lefebvre
**Coleoptera**	Mordellidae	Pascal Leblanc
	Malachiidae, Dasytidae	Robert Constantin & Vincent Lefebvre
	Cerambycidae, Cantharidae, Buprestidae, Rutelidae, Cetoniidae, Oedemeridae, and other Coleoptera	Vincent Lefebvre
**Hymenoptera**	**Apoidea**	
	Apidae – genus *Bombus*	Matthieu Aubert
	Apidae – genus *Nomada*	Eric Dufrêne
	Apidae – Other genera	Matthieu Aubert
	Halictidae	Matthieu Aubert & David Genoud
	Andrenidae	David Genoud
	Megachilidae	Matthieu Aubert
	Colletidae	Matthieu Aubert
	Ichneumonidae	Claire Villemant
	**Symphyta** – All families	David R. Smith
**Lepidoptera**	Nymphalidae, Zygaenidae, Lycaenidae	Jérôme Barbut, Emmanuel Delfosse, Axel Dehalleux, Vincent Lefebvre
**Other**	Heteroptera, Neuroptera	Armand Matocq, Vincent Lefebvre

Table S4: Anova table of the most parsimonious model testing the influence of altitude, Julian day (JD), their quadratic terms and interactions on the abundance of flowering-plants.

	SS	Df	F	Pr(>F)	
JD	68023	1	11.0417	0.0015	**
JD^2	73211	1	11.8838	<.0001	***
Altitude	36084	1	5.8572	0.0183	*
$Altitude^2$	52305	1	8.4902	0.0049	**
$JD:Altitude^2$	45641	1	7.4085	0.0083	**
Residuals	406600	66			

Table S5: Anova table of the most parsimonious model testing the influence of altitude, Julian day (JD), their quadratic terms and their interactions on the species richness of flowering-plants.

	SS	Df	F	Pr(>F)	
JD	21.841	1	20.005	<.0001	***
JD^2	21.823	1	19.988	<.0001	***
Altitude	14.367	1	13.159	<.0001	***
$Altitude^2$	22.137	1	20.275	<.0001	***
$JD:Altitude^2$	18.762	1	17.185	<.0001	***
Residuals	72.059	66			

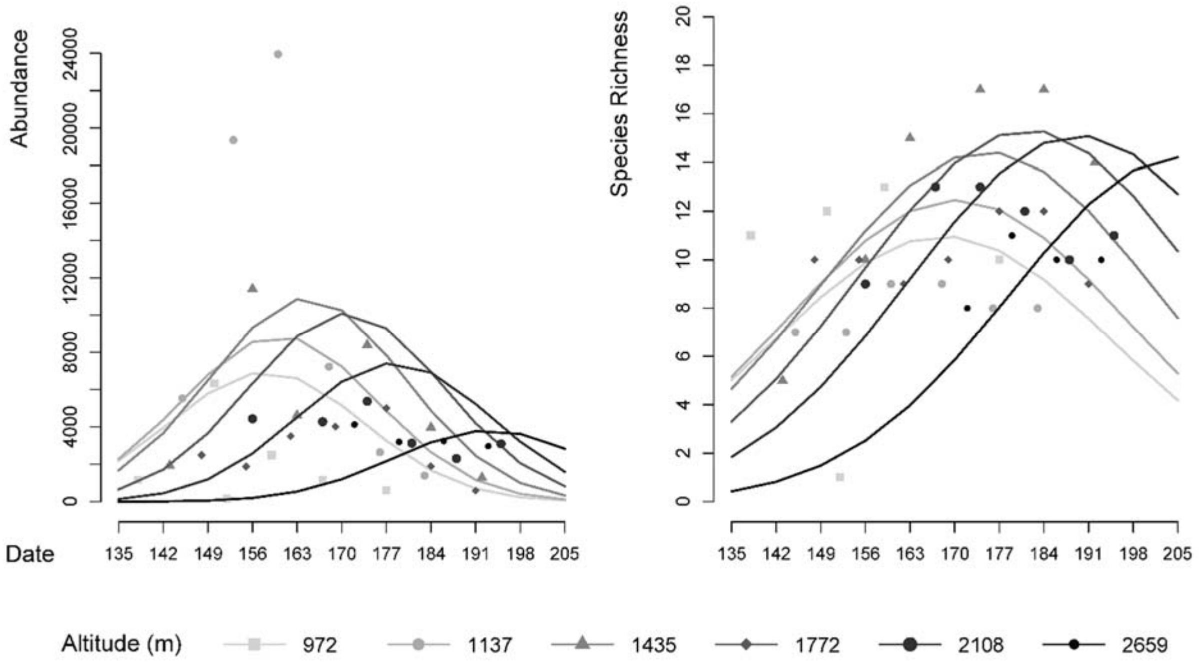

Figure S1: Abundance (left) and species richness (right) of flowering plants over time at six different elevations along the gradient. Lines represent the predictions of the most parsimonious models and dots the observed values for each day (4 x 60 = 240 m transects).

Table S6: Anova tables of the most parsimonious models testing the influence of insect order, altitude, Julian day (JD), their quadratic terms and interactions on the abundance (left) and species richness (right) of flower-visiting insects.

**Abundance**	SS	Df	F	Pr(>F)	**Species richness**	SS	Df	F	Pr(>F)
Order	410.73	3	13.1360	<.0001	Order	36.45	3	8.4512	<.0001
JD	246.05	1	23.6075	<.0001	JD	71.45	1	49.6959	<.0001
JD^2	311.57	1	29.8945	<.0001	JD^2	75.15	1	52.2644	<.0001
Altitude	34.29	1	3.2896	0.0709	Altitude	49.20	1	34.2213	<.0001
$Altitude^2$	795.24	1	76.3009	<.0001	$Altitude^2$	117.35	1	81.6154	<.0001
Order:JD^2	406.17	3	12.9903	<.0001	Order:JD^2	99.97	3	23.1768	<.0001
Order:$Altitude^2$	1034.81	3	33.0956	<.0001	Order:Altitude	269.43	3	62.4618	<.0001
JD:Altitude	292.63	1	28.0768	<.0001	JD:$Altitude^2$	82.16	1	57.1380	<.0001
Residuals	2761.94	265			Residuals	381.03	265		

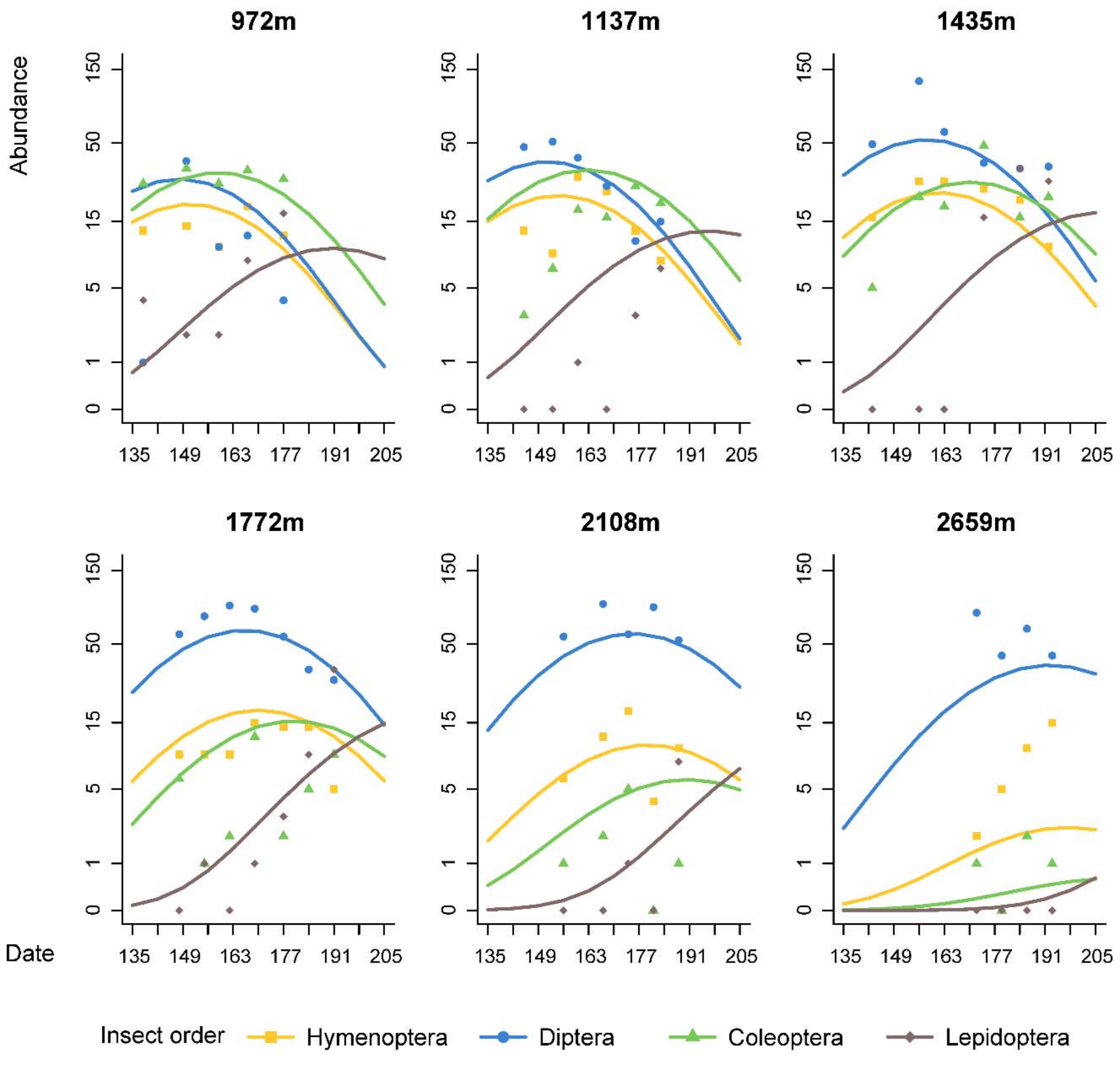

Figure S2: Abundances of the four major anthophilous orders over time at six different elevations along the gradient, without the honey bee *Apis mellifera*.

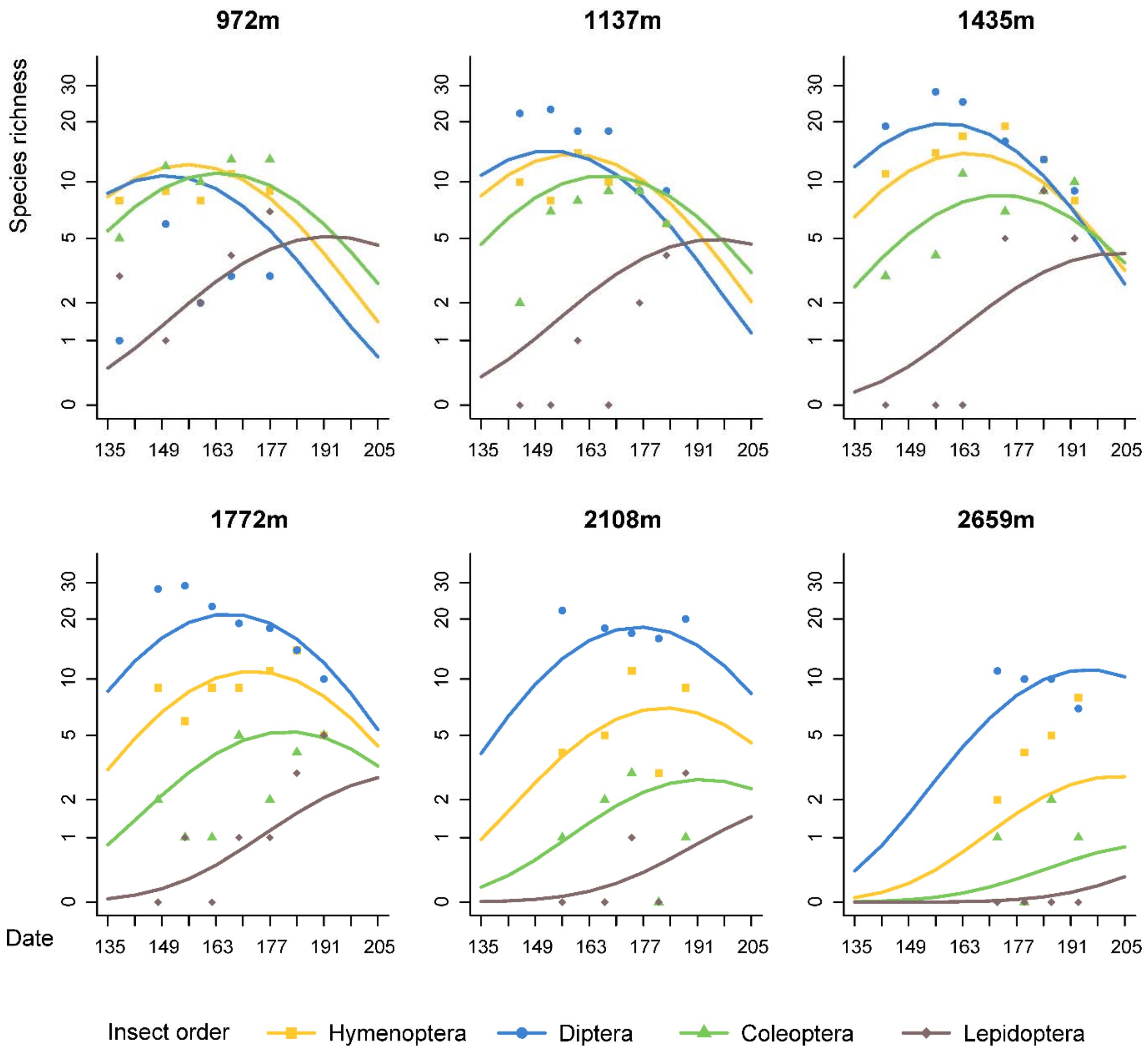

Figure S3: Species richness of the four major anthophilous orders over time at six different elevations along the gradient. Lines represent the predictions of the most parsimonious models and dots the observed values for each day (4 x60 m transects).

Table S7: P-values for Jost's tests examining the significance of the Bray-Curtis dissimilarities in the choice of plant species visited among orders along the gradient.

	Coleoptera	Diptera	Hymenoptera
Coleoptera		2.03e-05	1.08e-05
Diptera			2.91e-06
Hymenoptera			

Table S8: Anova tables of the most parsimonious models testing the effects of insect family, altitude and its quadratic term, Julian day (JD) and its quadratic term, and interactions on the abundance (left) and species richness (right) of flower-visiting flies.

**Abundance**					**Species richness**				
	SS	Df	F	Pr(>F)		SS	Df	F	Pr(>F)
Family	74.91	3	3.0954	0.0274	Family	52.75	3	12.6030	<.0001
JD	135.61	1	16.8114	<.0001	JD	16.57	1	11.8760	0.0006
JD^2	166.68	1	20.6626	<.0001	JD^2	20.05	1	14.3754	0.0002
Altitude	8.54	1	1.0590	0.3044	Altitude	7.63	1	5.4664	0.0201
$Altitude^2$	0.01	1	0.0006	0.9798	$Altitude^2$	52.35	1	37.5266	<.0001
Family:JD^2	141.73	3	5.8567	0.0007	Family:JD	34.48	3	8.2388	<.0001
Family:Altitude	68.43	3	2.8278	0.0390	Family:$Altitude^2$	48.09	3	11.4908	<.0001
Family:$Altitude^2$	95.76	3	3.9569	0.0087	JD^2:Altitude	7.97	1	5.7115	0.0176
JD^2:Altitude	107.30	1	13.3012	0.0003	JD:Altitude	5.71	1	4.0908	0.0441
Residuals	2113.50	262			Residuals	368.29	264		

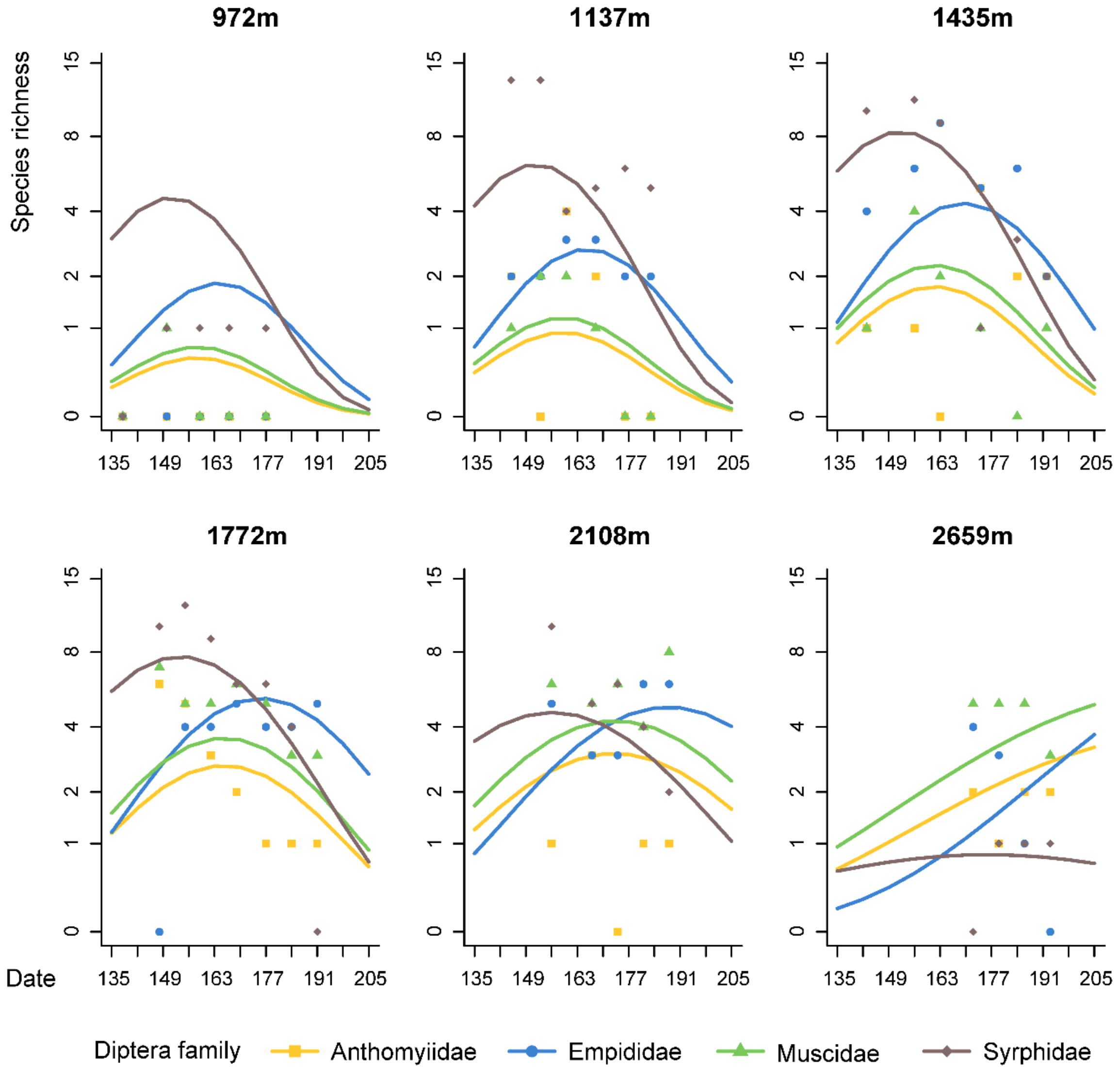

Figure S4: Species richness of the four major dipteran families over time at six different elevations along the gradient.

Table S9: P-values for Jost's tests examining the significance of the Bray-Curtis dissimilarities in the choice of plants species visited among fly families along the gradient.

	Syrphidae	Empididae	Muscidae	Anthomyiidae
Syrphidae		<0.0001	<0.0001	0,0991
Empididae			<0.0001	0,0375
Muscidae				0,0261
Anthomyiidae				

Table S10: Anova tables of the most parsimonious models testing the influence of insect family, altitude and its quadratic term, Julian day (JD) and its quadratic term, and interactions on the abundance and species richness of flower visiting hymenopterans.

**Abundance**					**Species richness**				
	SS	Df	F	Pr(>F)		SS	Df	F	Pr(>F)
Family	67.74	3	3.5205	0.0156	Family	85.188	3	25.5679	<.0001
JD	12.89	1	2.0094	0.1575	JD	2.431	1	2.1887	0.1402
JD²	17.68	1	2.7569	0.0980	JD²	0.064	1	0.0578	0.8102
Altitude	151.87	1	23.6794	<.0001	Altitude	8.521	1	7.6726	0.0060
Altitude²	151.34	1	23.5954	<.0001	Altitude²	9.323	1	8.3948	0.0041
Family:JD	73.06	3	3.7971	0.0108	Family:Altitude²	31.983	3	9.5991	<.0001
Family:JD²	76.32	3	3.9665	0.0086	JD²:Altitude	5.283	1	4.7571	0.0300
Family:Altitude²	57.39	3	2.9828	0.0318	JD:Altitude²	7.600	1	6.8427	0.0094
JD:Altitude²	103.34	1	16.1117	<.0001	Residuals	296.534	267		
Residuals	1680.41	262							

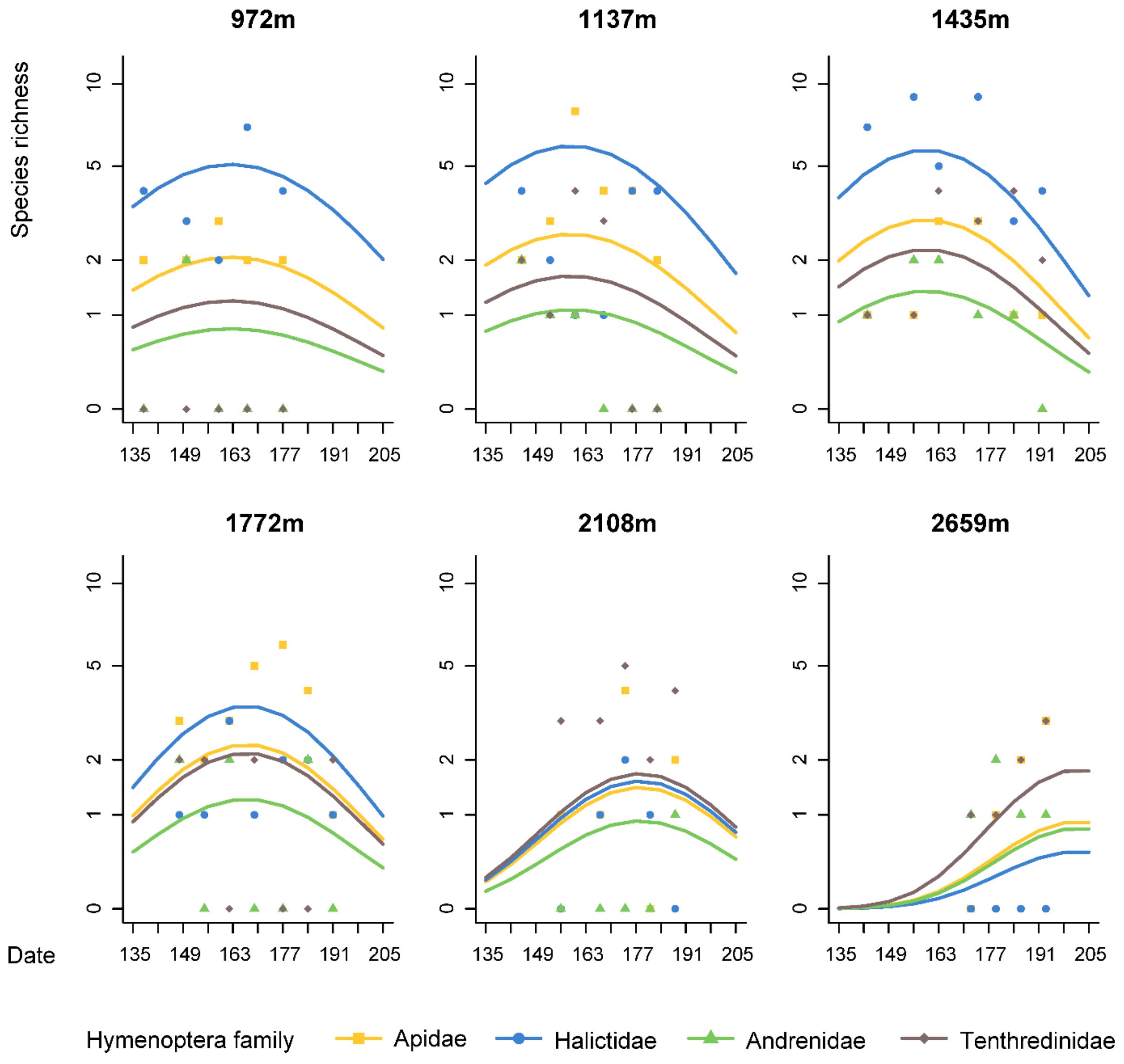

Figure S5: Species richness of the four major hymenopteran families over time at six different elevations along the gradient.

Table S11: Anova table of the most parsimonious model testing the influence of insect family, altitude and its quadratic term, Julian day (JD) and its quadratic term, and interactions on the abundance of flower visiting hymenopterans excluding *Apis mellifera*.

	SS	Df	F	Pr(>F)	
Family	39.06	3	3.8599	0.0010	**
JD	5.07	1	1.5021	0.2214	
JD^2	1.53	1	0.4541	0.5010	
Altitude	23.39	1	6.9350	0.0089	**
$Altitude^2$	30.49	1	9.0385	0.0029	**
Family:JD	38.52	3	3.8060	0.0107	*
Family:JD^2	39.59	3	3.9125	0.0093	**
Family:$Altitude^2$	45.25	3	4.4710	0.0044	**
JD^2:Altitude	16.25	1	4.8168	0.0291	*
JD:$Altitude^2$	26.13	1	7.7458	0.0058	**
Residuals	880.44	261			

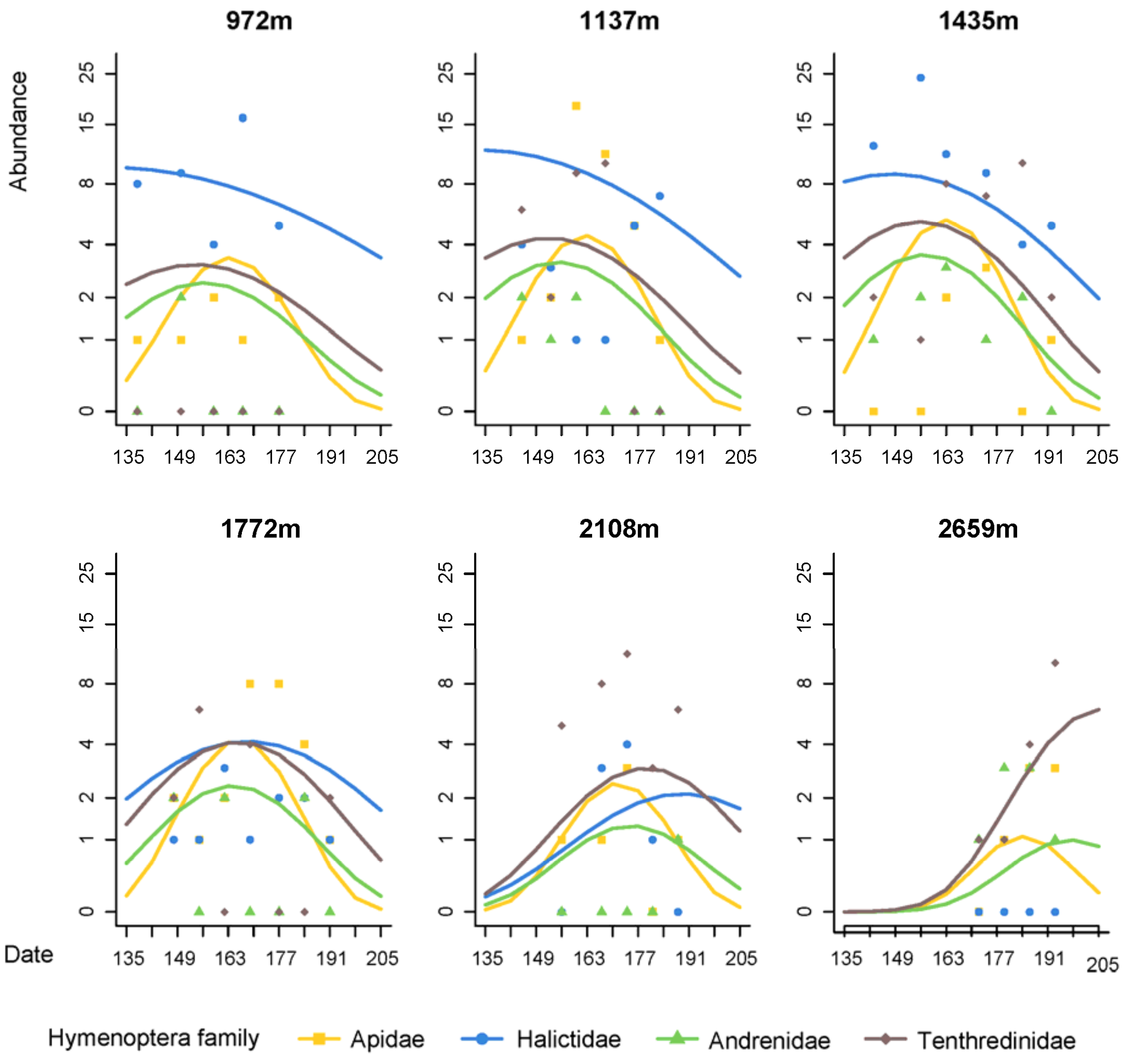

Figure S6: Abundances of the four major hymenopteran families over time at six different elevations along the gradient, without *Apis mellifera*.

Table S12: Anova tables of the most parsimonious models testing the influence of insect family, altitude and its quadratic term, Julian day (JD) and its quadratic term, and interactions on the abundance (left) and species richness (right) of flower visiting beetles.

	**Abundance**					**Species richness**				
	SS	Df	F	Pr(>F)		SS	Df	F	Pr(>F)	
Family	146.00	4	11.9854	<.0001	***	10.017	4	5.3780	0.0003	***
JD	15.28	1	5.0179	0.0258	*	2.065	1	4.4345	0.0360	*
JD^2	19.96	1	6.5548	0.0109	*	2.575	1	5.5298	0.0193	*
Altitude	2.26	1	0.7408	0.3901		0.369	1	0.7927	0.3739	
$Altitude^2$	15.23	1	5.0016	0.0260	*	0.946	1	2.0316	0.1550	
Family:JD	130.15	4	10.6840	<.0001	***	7.483	4	4.0174	0.0034	**
Family:JD^2	147.51	4	12.1090	<.0001	***	7.485	4	4.0188	0.0034	**
Family:Altitude	171.15	4	14.0492	<.0001	***	10.205	4	5.4791	0.0003	***
Family:$Altitude^2$	95.08	4	7.8047	<.0001	***	16.192	4	8.6936	<.0001	***
JD^2:Altitude	5.21	1	1.7121	0.1917		0.284	1	0.6093	0.4356	
JD:Altitude	0.00	1	0.0000	0.9970		0.031	1	0.0660	0.7974	
JD:$Altitude^2$	28.96	1	9.5101	0.0022	**	2.279	1	4.8951	0.0276	*
Family:JD:Altitude	89.01	4	7.3066	<.0001	***	5.274	4	2.8314	0.0248	*
Residuals	968.46	318				148.074	318			

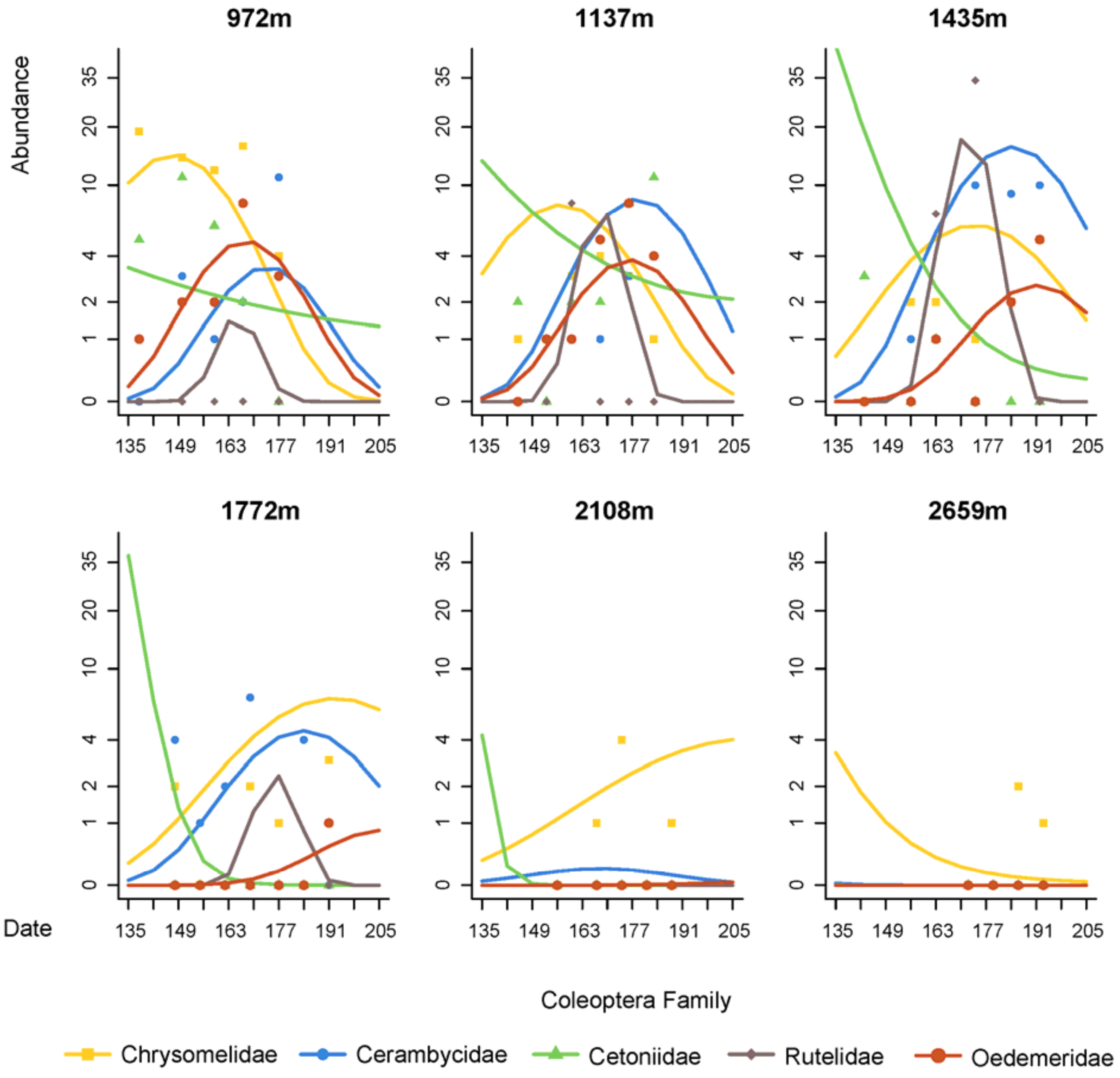

Figure S7: Abundances of the five major coleopteran families over time at six different elevations along the gradient.

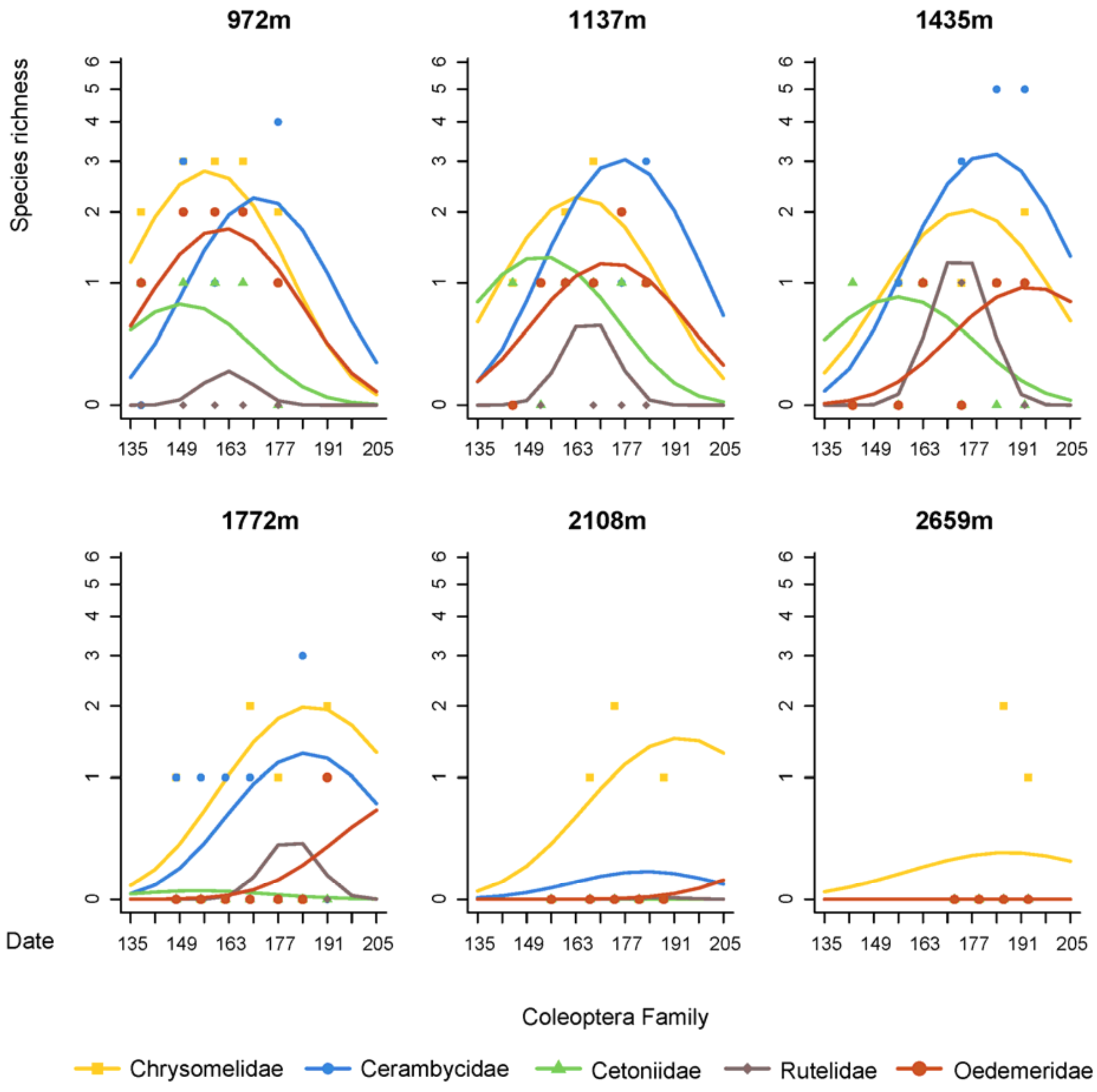

Figure S8: Species richness of the four major coleopteran families over time at six different elevations along the gradient.

Table S13: Anova table of the most parsimonious model testing the influence of insect family, altitude and its quadratic term, Julian day (JD) and its quadratic term, and interactions on the abundance of flower-visiting lepidopterans.

	SS	Df	F	Pr(>F)	
Family	53.13	3	3.1050	0.0270	*
JD	54.64	1	9.5803	0.0022	**
JD^2	62.64	1	10.9837	0.0010	**
Altitude	4.38	1	0.7682	0.3815	
$Altitude^2$	93.01	1	16.3085	<.0001	***
Family:Altitude	32.38	1	5.6773	0.0179	*
Residuals	1545.55	271			

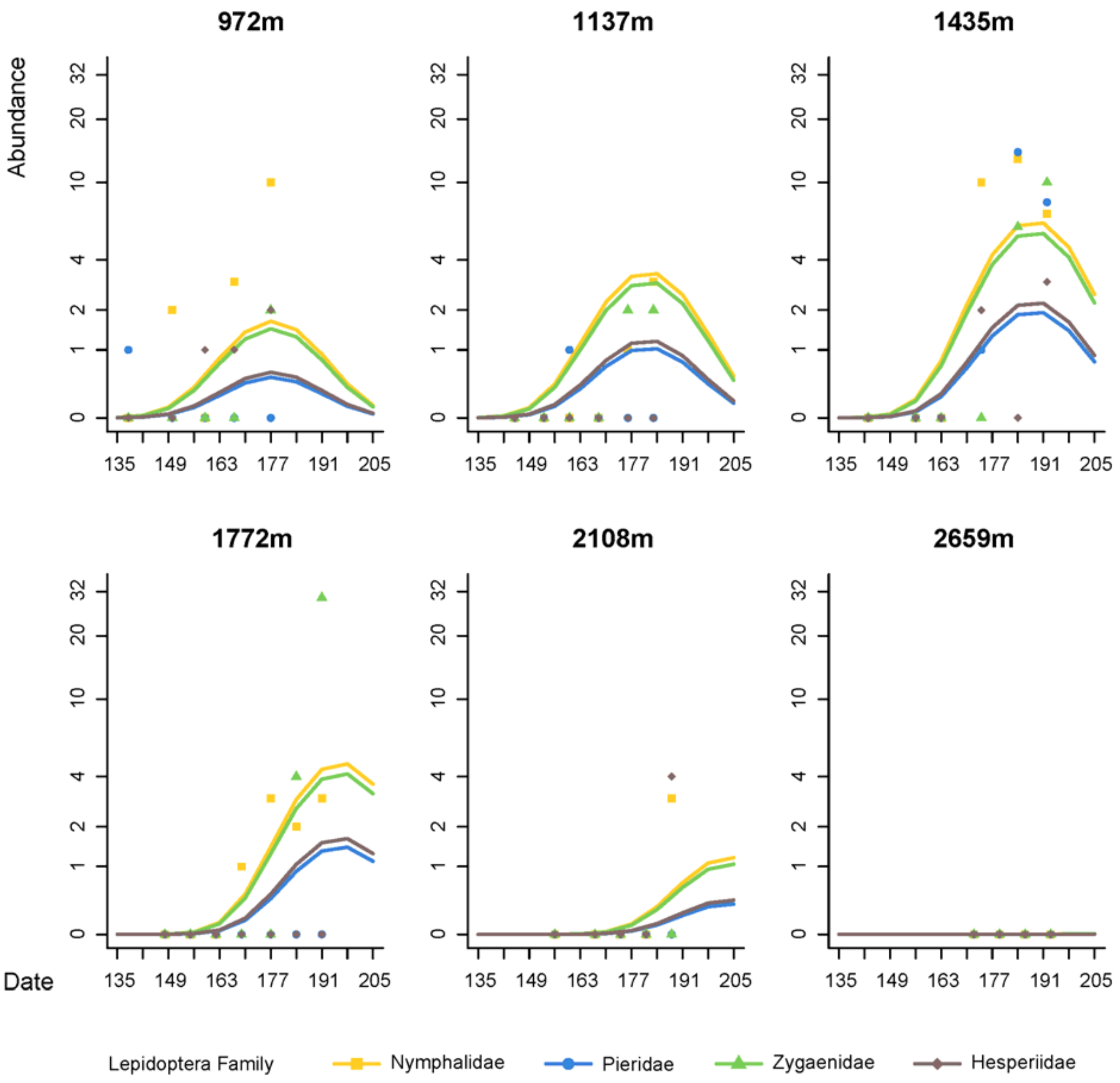

Figure S9: Abundances of the four major lepidopteran families over time at six different elevations along the gradient.

Table S14: Anova table of the most parsimonious model testing the influence of insect family, altitude and its quadratic term, Julian day (JD) and its quadratic term, and interactions on the species richness of flower-visiting lepidopterans.

	SS	Df	F	Pr(>F)	
Family	0.465	3	0.1976	0.89797	
JD	1.381	1	1.7590	0.18594	
JD²	1.664	1	2.1187	0.14674	
Altitude	1.795	1	2.2862	0.13177	
Altitude²	3.231	1	4.1142	0.04357	*
Family:JD	1.179	3	0.5005	0.68227	
Family:JD²	0.860	3	0.3653	0.77815	
Family:Altitude	5.533	3	2.3489	0.07304	.
Family:Altitude²	6.563	3	2.7859	0.04131	*
JD²:Altitude	4.183	1	5.3271	0.02180	*
JD:Altitude	3.567	1	4.5426	0.03402	*
JD:Altitude²	2.922	1	3.7215	0.05483	.
Family:JD:Altitude²	6.955	3	2.9524	0.03320	*
Residuals	199.443	254			

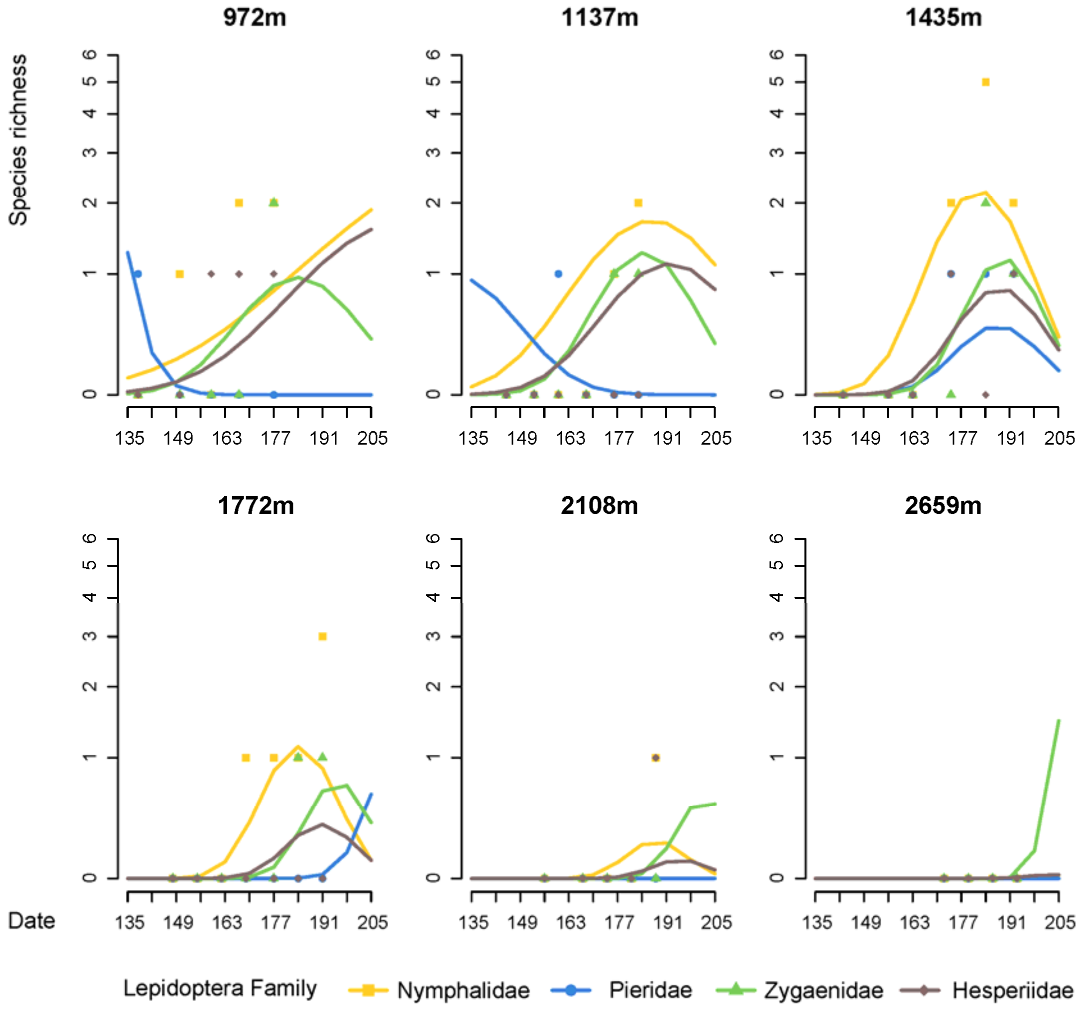

Figure S10: Species richness of the four major lepidopteran families over time at six different elevations along the gradient.

Table S15. Species of Angiosperm recorded along the transects with their pollination regime. ento = entomophilous; anemo = anemogamous; apo = apogamous; auto = autogamous (Tela Botanica, 2018).

Code	Family	Taxon	Pollination
**F001**	Asteraceae	*Achillea millefolium* L., 1753	ento
**F002**	Lamiaceae	*Ajuga genevensis* L., 1753	ento
**F003**	Lamiaceae	*Ajuga pyramidalis* L., 1753	ento-auto
**F004**	Amaryllidaceae	*Allium schoenoprasum* L., 1753	ento-auto
**F005**	Orchidaceae	*Anacamptis pyramidalis* (L.) Rich.,	ento
**F006**	Primulaceae	*Androsace adfinis* subsp. *brigantiaca* (Jord. & Fourr.) Kress, 1981	ento
**F007**	Ranunculaceae	*Anemone narcissiflora* L., 1753	ento
**F008**	Asteraceae	*Antennaria dioica* (L.) Gaertn., 1791	ento-apo
**F009**	Apiaceae	*Anthriscus sylvestris* (L.) Hoffm., 1814	ento
**F010**	Fabaceae	*Anthyllis vulneraria* subsp. *alpestris* (Kit.) Asch. & Graebn., 1908	ento
**F011**	Fabaceae	*Anthyllis vulneraria* subsp. *polyphylla* (DC.) Nyman, 1878	ento
**F012**	Ranunculaceae	*Aquilegia vulgaris* L., 1753	ento
**F013**	Plumbaginaceae	*Armeria alpina* Willd., 1809	ento
**F014**	Asteraceae	*Aster alpinus* L. subsp. *alpinus*	ento-auto
**F015**	Fabaceae	*Astragalus danicus* Retz., 1783	ento
**F016**	Fabaceae	*Astragalus monspessulanus* L. subsp. *monspessulanus*	ento
**F017**	Brassicaceae	*Biscutella laevigata* L., 1771	ento
**F018**	Convolvulaceae	*Calystegia sepium* (L.) R.Br., 1810	ento
**F019**	Campanulaceae	*Campanula scheuchzeri*	ento-auto
**F020**	Asteraceae	*Carduus nutans* L., 1753	ento-auto
**F021**	Apiaceae	*Carum carvi* L., 1753	ento
**F022**	Apiaceae	*Bunium* sp.	ento
**F023**	Asteraceae	*Centaurea jacea* L. subsp. *jacea*	ento-auto
**F024**	Asteraceae	*Centaurea scabiosa* L., 1753	ento-auto
**F025**	Asteraceae	*Centaurea uniflora* Turra subsp. *uniflora*	ento-auto
**F026**	Caryophyllaceae	*Cerastium* sp.	ento
**F027**	Boraginaceae	*Cerinthe minor* subsp. *auriculata* (Ten.) Rouy, 1927	ento
**F028**	Asteraceae	*Cyanus montanus* (L.) Hill, 1768	ento
**F029**	Asteraceae	*Cyanus segetum* Hill, 1762	ento-auto
**F030**	Caryophyllaceae	*Dianthus pavonius* Tausch, 1839	ento
**F031**	Brassicaceae	*Draba aizoides* L., 1767	ento
**F032**	Brassicaceae	*Erysimum* sp.	ento
**F033**	Euphorbiaceae	*Euphorbia cyparissias* L., 1753	ento
**F034**	Rubiaceae	*Galium glaucum* L., 1753	ento
**F035**	Rubiaceae	*Galium verum* L., 1753	ento-auto
**F036**	Gentianaceae	*Gentiana acaulis* L., 1753	ento
**F037**	Gentianaceae	*Gentiana lutea* L., 1753	ento
**F038**	Gentianaceae	*Gentiana verna* L., 1753	ento
**F039**	Gentianaceae	*Gentianella campestris* (L.) Börner, 1912	ento
**F040**	Geraniaceae	*Geranium pyrenaicum* Burm.f., 1759	ento-auto

**F041**	Geraniaceae	*Geranium sylvaticum* L., 1753	ento
**F042**	Rosaceae	*Geum montanum* L., 1753	ento
**F043**	Orchidaceae	*Gymnadenia conopsea* (L.) R.Br., 1813	ento
**F044**	Orchidaceae	*Gymnadenia nigra* subsp. *corneliana* (Beauverd) J.M.Tison, 2010	ento
**F045**	Cistaceae	*Helianthemum nummularium* (L.) Mill., 1768	ento-auto
**F046**	Asteraceae	*Hieracium cymosum* L., 1763	apo
**F047**	Fabaceae	*Hippocrepis comosa* L., 1753	ento
**F048**	Asteraceae	*Hypochaeris maculata* L., 1753	ento
**F049**	Caprifoliaceae	*Knautia arvensis* (L.) Coult., 1828	ento
**F050**	Fabaceae	*Lathyrus pratensis* L., 1753	ento-auto
**F051**	Asteraceae	*Leontodon hispidus* L. subsp. *hispidus*	ento-auto
**F052**	Asteraceae	*Leontopodium nivale* subsp. *alpinum* (Cass.) Greuter, 2003	ento-auto
**F053**	Asteraceae	*Leucanthemopsis alpina* (L.) Heywood, 1975	ento-auto
**F054**	Asteraceae	*Leucanthemum coronopifolium* (Vill.) Horvatic, 1935	ento-auto
**F055**	Asteraceae	*Leucanthemum vulgare* Lam., 1779	ento-auto
**F056**	Linaceae	*Linum alpinum* Jacq., 1762	ento
**F057**	Fabaceae	*Lotus* sp.	ento
**F058**	Fabaceae	*Medicago lupulina* L., 1753	ento
**F059**	Fabaceae	*Melilotus officinalis* (L.) Lam., 1779	ento
**F060**	Apiaceae	*Meum athamanticum* Jacq., 1776	ento
**F061**	Boraginaceae	*Myosotis alpestris* F.W.Schmidt, 1794	ento
**F062**	Boraginaceae	*Myosotis decumbens* Host, 1827	ento
**F063**	Amaryllidaceae	*Narcissus poeticus* L., 1753	ento
**F064**	Fabaceae	*Onobrychis viciifolia* [subsp. *montana* (DC.) Gams, 1924]	ento
**F065**	Asparagaceae	*Ornithogalum umbellatum* L., 1753 S.str.	ento-auto
**F066**	Orobanchaceae	*Pedicularis gyroflexa* Vill., 1785	ento
**F067**	Orobanchaceae	*Pedicularis rostratospicata* subsp. *helvetica* (Steininger) O.Schwarz, 1949	ento
**F068**	Orobanchaceae	*Pedicularis tuberosa* L., 1753	ento
**F069**	Polygonaceae	*Persicaria bistorta* (L.) Samp., 1913	ento
**F070**	Campanulaceae	*Phyteuma betonicifolium* Vill., 1785	ento
**F071**	Campanulaceae	*Phyteuma orbiculare* L., 1753	ento
**F073**	Asteraceae	*Picris hieracioides* L., 1753	ento-auto
**F074**	Asteraceae	*Pilosella lactucella* (Wallr.) P.D.Sell & C.West, 1967	apo
**F075**	Plantaginaceae	*Plantago lanceolata* L., 1753	ento-anemo
**F076**	Plantaginaceae	*Plantago major* L., 1753	anemo
**F077**	Polygalaceae	*Polygala* sp.	ento
**F078**	Rosaceae	*Potentilla aurea* L., 1756	ento
**F079**	Rosaceae	*Potentilla crantzii* (Crantz) Beck ex Fritsch, 1897	apo
**F080**	Primulaceae	*Primula veris* L., 1753	ento
**F081**	Ranunculaceae	*Ranunculus bulbosus* L., 1753	ento
**F082**	Ranunculaceae	*Ranunculus kuepferi* Greuter & Burdet, 1987	ento
**F083**	Ranunculaceae	*Ranunculus montanus* Willd., 1799	ento
**F084**	Orobanchaceae	*Rhinanthus alectorolophus* (Scop.) Pollich, 1777	ento
**F085**	Lamiaceae	*Salvia pratensis* L., 1753	ento

**F086**	Caryophyllaceae	*Saponaria ocymoides* L., 1753	ento
**F087**	Saxifragaceae	*Saxifraga exarata* Vill. subsp. *exarata*	ento
**F088**	Asteraceae	*Scorzonera hispanica* L., 1753	ento
**F089**	Crassulaceae	*Sempervivum arachnoideum* L., 1753	ento
**F090**	Crassulaceae	*Sempervivum montanum* L., 1753	ento
**F091**	Asteraceae	*Senecio doronicum* (L.) L. subsp. *doronicum*	ento-auto
**F092**	Caryophyllaceae	*Silene latifolia* subsp. *alba* (Mill.) Greuter & Burdet, 1982	ento
**F093**	Primulaceae	*Soldanella alpina* L., 1753	ento
**F094**	Lamiaceae	*Stachys recta* L., 1767	ento
**F095**	Asteraceae	*Taraxacum officinale (gr.)*	apo
**F096**	Asteraceae	*Tragopogon pratensis* L., 1753	ento-auto
**F097**	Fabaceae	*Trifolium alpinum* L., 1753	ento
**F098**	Fabaceae	*Trifolium montanum* L. subsp. *montanum*	ento
**F099**	Fabaceae	*Trifolium pratense* L. subsp. *pratense*	ento
**F100**	Fabaceae	*Trifolium repens* L., 1753	ento
**F101**	Ranunculaceae	*Trollius europaeus* L., 1753	ento-auto
**F102**	Caprifoliaceae	*Valeriana* sp.	ento
**F103**	Fabaceae	*Vicia tenuifolia* Roth subsp. t*enuifolia*	ento
**F104**	Violaceae	*Viola tricolor* L., 1753	ento
**F105**	Caprifoliaceae	*Valerianella* sp.	auto
**F106**	Boraginaceae	*Echium vulgare* L., 1753	ento
**F107**	Orchidaceae	*Neotinea ustulata* (L.) Bateman, Pridgeon & Chase subsp. *ustulata*	ento
**F108**	Polygonaceae	*Persicaria vivipara* (L.) Ronse Decr., 1988	ento
**F109**	Rosaceae	*Potentilla* sp.	ento
**F110**	Campanulaceae	*Campanula* sp. [R2100]	ento-auto
**F111**	Fabaceae	*Lotus corniculatus* L., 1753	ento
**F112**	Rosaceae	*Geum rivale* L., 1753	ento-auto
**F113**	Fabaceae	*Lotus alpinus* (DC.) Schleich. ex Ramond, 1825	ento
**F114**	Fabaceae	*Trifolium badium* Schreb., 1804	ento
**F115**	Xanthorrhoeaceae	*Paradisea liliastrum* (L.) Bertol., 1840	ento
**F116**	Caryophyllaceae	*Silene acaulis* (L.) Jacq. subsp. *acaulis*	ento
**F117**	Primulaceae	*Androsace vitaliana* (L.) Lapeyr., 1813	ento
**F118**	Asteraceae	*Erigeron uniflorus* L., 1753	ento-auto
**F119**	Asteraceae	*Cirsium spinosissimum* (L.) Scop., 1769	ento-auto
**F120**	Asteraceae	*Doronicum grandiflorum* Lam., 1786	ento
**F121**	Fabaceae	*Trifolium thalii* Vill., 1779	ento
**F122**	Asteraceae	*Achillea nana* L., 1753	ento

References

Tela Botanica, 2018. Tela Botanica. http://www.tela-botanica.org/. (Accessed 20 January 2018).

Table S16. Insect taxa collected on flowers or identified by sight along the transects.

Ordre	Famille	Taxon
Coleoptera	Buprestidae	Acmaeoderella flavofasciata (Piller & Mitterpacher, 1783)
Coleoptera	Buprestidae	Anthaxia fulgurans (Schrank, 1789)
Coleoptera	Buprestidae	Anthaxia helvetica Stierlin, 1868
Coleoptera	Buprestidae	Anthaxia nitidula (Linnaeus, 1758)
Coleoptera	Buprestidae	Anthaxia quadripunctata (Linnaeus, 1758)
Coleoptera	Buprestidae	Anthaxia semicruprea Küster, 1851
Coleoptera	Byturidae	Byturus ochraceus (Scriba, 1790)
Coleoptera	Cantharidae	Cantharis annularis Ménétriés, 1836
Coleoptera	Cantharidae	Cantharis lateralis Linnaeus, 1758
Coleoptera	Cantharidae	Cantharis livida Goeze, 1777
Coleoptera	Cantharidae	Cantharis nigra (De Geer, 1774)
Coleoptera	Cantharidae	Cantharis nigricans (O.F. Müller, 1776)
Coleoptera	Cantharidae	Cantharis pallida Goeze, 1777
Coleoptera	Cantharidae	Cantharis rustica Fallén, 1807
Coleoptera	Cantharidae	Cantharis sp.
Coleoptera	Cantharidae	Cantharis tristis Fabricius, 1798
Coleoptera	Cantharidae	Rhagonycha fulva (Scopoli, 1763)
Coleoptera	Carabidae	Harpalus sp.
Coleoptera	Cerambycidae	Alosterna tabacicolor (De Geer, 1775)
Coleoptera	Cerambycidae	Anastrangalia dubia (Scopoli, 1763)
Coleoptera	Cerambycidae	Anastrangalia sanguinolenta (Linnaeus, 1760)
Coleoptera	Cerambycidae	Brachyta interrogationis (Linnaeus, 1758)
Coleoptera	Cerambycidae	Deilus fugax (Olivier, 1790)
Coleoptera	Cerambycidae	Dinoptera collaris (Linnaeus, 1758)
Coleoptera	Cerambycidae	Grammoptera ruficornis (Fabricius, 1781)
Coleoptera	Cerambycidae	Pachytodes cerambyciformis (Schrank, 1781)
Coleoptera	Cerambycidae	Paracorymbia hybrida (Rey, 1885)
Coleoptera	Cerambycidae	Paracorymbia maculicornis (De Geer, 1775)
Coleoptera	Cerambycidae	Pseudovadonia livida (Fabricius, 1777)
Coleoptera	Cerambycidae	Rutpela maculata (Poda, 1761)
Coleoptera	Cerambycidae	Stenopterus rufus Linnaeus, 1767
Coleoptera	Cerambycidae	Stenurella bifasciata (Müller, 1776)
Coleoptera	Cerambycidae	Stenurella melanura Linnaeus, 1758
Coleoptera	Cerambycidae	Stenurella nigra (Linnaeus, 1758)
Coleoptera	Cetoniidae	Cetonia aurata (Linnaeus, 1761)
Coleoptera	Cetoniidae	Oxythyrea funesta (Poda, 1761)
Coleoptera	Cetoniidae	Trichius fasciatus (Linnaeus, 1758)
Coleoptera	Cetoniidae	Tropinota hirta (Poda, 1761)
Coleoptera	Cetoniidae	Valgus hemipterus (Linnaeus, 1758)
Coleoptera	Chrysomelidae	Bruchidae sp.
Coleoptera	Chrysomelidae	Cassida sp.

Coleoptera	Chrysomelidae	Clytra quadripunctata (Linnaeus, 1758)
Coleoptera	Chrysomelidae	Cryptocephalus aureolus Suffrian, 1847
Coleoptera	Chrysomelidae	Cryptocephalus bipunctatus (Linnaeus, 1758)
Coleoptera	Chrysomelidae	Cryptocephalus carinthiacus Suffrian, 1848
Coleoptera	Chrysomelidae	Cryptocephalus globicollis Suffrian, 1847
Coleoptera	Chrysomelidae	Cryptocephalus gr. sericeus
Coleoptera	Chrysomelidae	Cryptocephalus hypochaeridis (Linnaeus, 1758)
Coleoptera	Chrysomelidae	Cryptocephalus sericeus (Linnaeus, 1758)
Coleoptera	Chrysomelidae	Cryptocephalus sp.
Coleoptera	Chrysomelidae	Cryptocephalus violaceus Laicharting, 1781
Coleoptera	Chrysomelidae	Labidostomis longimana (Linnaeus, 1760)
Coleoptera	Chrysomelidae	Luperus sp.
Coleoptera	Chrysomelidae	Neocrepidodera peirolerii (Kutschera, 1860)
Coleoptera	Chrysomelidae	Oreina collucens (J. Daniel, 1903)
Coleoptera	Cleridae	Trichodes apiarius (Linnaeus, 1758)
Coleoptera	Cleridae	Trichodes leucopsideus (Olivier, 1795)
Coleoptera	Curculionidae	Miarus graminis (Gyllenhal, 1813)
Coleoptera	Curculionidae	Miarus sp.
Coleoptera	Curculionidae	Phyllobius pyri (Linnaeus, 1758)
Coleoptera	Curculionidae	Zacladus exiguus (Olivier, 1807)
Coleoptera	Dascillidae	Dascillus cervinus (Linnaeus, 1758)
Coleoptera	Dasytidae	Aplocnemus alpestris/virens
Coleoptera	Dasytidae	Dasytes gonocerus Mulsant & Rey, 1868
Coleoptera	Dasytidae	Dasytes plumbeus (O.F. Müller, 1776)
Coleoptera	Dasytidae	Dasytes subalpinus Baudi di Selve, 1874
Coleoptera	Dasytidae	Dasytidae sp.
Coleoptera	Dermestidae	Anthrenus verbasci (Linnaeus, 1767)
Coleoptera	Elateridae	Agriotes sp.
Coleoptera	Elateridae	Agrypnus murinus (Linnaeus, 1758)
Coleoptera	Elateridae	Cidnopus pilosus (Leske, 1785)
Coleoptera	Elateridae	Ctenicera cuprea (Fabricius, 1775)
Coleoptera	Elateridae	Limonius minutus (Linnaeus, 1758)
Coleoptera	Elateridae	Limonius sp.
Coleoptera	Elateridae	Prosternon tesselatum (Linnaeus, 1758)
Coleoptera	Malachiidae	Clanoptilus arnaizi (Pardo Alcaide, 1966)
Coleoptera	Malachiidae	Clanoptilus barnevillei (Puton, 1865)
Coleoptera	Malachiidae	Clanoptilus elegans (Olivier, 1790)
Coleoptera	Malachiidae	Cordylepherus viridis (Fabricius, 1787)
Coleoptera	Malachiidae	Malachius aeneus (Linnaeus, 1758)
Coleoptera	Malachiidae	Malachius australis Mulsant & Rey, 1867
Coleoptera	Malachiidae	Malachius bipustulatus (Linnaeus, 1758)
Coleoptera	Malachiidae	Micrinus inornatus (Küster, 1846)
Coleoptera	Meloidae	Hycleus polymorphus (Pallas, 1771)
Coleoptera	Meloidae	Mylabris variabilis (Pallas, 1781)

Coleoptera	Mordellidae	Mordella holomelaena Apfelbeck, 1914
Coleoptera	Mordellidae	Mordellistena brevicauda (Boheman, 1849)
Coleoptera	Mordellidae	Mordellistena pseudopumila Ermisch, 1963
Coleoptera	Mordellidae	Mordellistena sp.
Coleoptera	Mordellidae	Mordellochroa abdominalis (Fabricius, 1775)
Coleoptera	Nitidulidae	Meligethes sp.
Coleoptera	Nitidulidae	Meligethes sp.1
Coleoptera	Nitidulidae	Meligethes sp.2
Coleoptera	Oedemeridae	Chrysanthia viridissima (Linnaeus, 1758)
Coleoptera	Oedemeridae	Oedemera femorata (Scopoli, 1763)
Coleoptera	Oedemeridae	Oedemera flavipes (Fabricius, 1792)
Coleoptera	Oedemeridae	Oedemera lurida (Marsham, 1802)
Coleoptera	Oedemeridae	Oedemera podagrariae (Linnaeus, 1767)
Coleoptera	Oedemeridae	Oedemera virescens (Linnaeus, 1767)
Coleoptera	Phalacridae	Olibrus sp.
Coleoptera	Rutelidae	Phyllopertha horticola (Linnaeus, 1758)
Coleoptera	Staphylinidae	Staphylinidae sp.
Diptera	Agromyzidae	Agromyzidae sp.
Diptera	Anthomyiidae	Adia cinerella (Fallén, 1825)
Diptera	Anthomyiidae	Adia coerulescens (Strobl, 1893)
Diptera	Anthomyiidae	Adia grisella (Rondani, 1871)
Diptera	Anthomyiidae	Anthomyiidae sp.
Diptera	Anthomyiidae	Anthomyiidae sp.10
Diptera	Anthomyiidae	Anthomyiidae sp.2
Diptera	Anthomyiidae	Anthomyiidae sp.3
Diptera	Anthomyiidae	Anthomyiidae sp.4
Diptera	Anthomyiidae	Anthomyiidae sp.5
Diptera	Anthomyiidae	Anthomyiidae sp.6
Diptera	Anthomyiidae	Anthomyiidae sp.7
Diptera	Anthomyiidae	Anthomyiidae sp.8
Diptera	Anthomyiidae	Anthomyiidae sp.9
Diptera	Anthomyiidae	Botanophila striolata (Fallén, 1824)
Diptera	Anthomyiidae	Botanophila varicolor (Meigen, 1826)
Diptera	Anthomyiidae	Chirosia sp.
Diptera	Anthomyiidae	Delia platura (Meigen, 1826)
Diptera	Anthomyiidae	Delia sp.1
Diptera	Anthomyiidae	Delia sp.2
Diptera	Anthomyiidae	Egle sp.
Diptera	Anthomyiidae	Hylemya variata (Fallén, 1823)
Diptera	Anthomyiidae	Paregle audacula (Harris, 1780)
Diptera	Anthomyiidae	Paregle sp.
Diptera	Anthomyiidae	Pegoplata aestiva (Meigen, 1826)
Diptera	Anthomyiidae	Phorbia sp.
Diptera	Asilidae	Asilidae sp.

Diptera	Bibionidae	Bibio hortulanus (Linnaeus, 1758)
Diptera	Bibionidae	Bibionidae sp.
Diptera	Bombyliidae	Bombylella atra (Scopoli, 1763)
Diptera	Bombyliidae	Bombyliidae sp.
Diptera	Bombyliidae	Bombylius sp.
Diptera	Bombyliidae	Lomatia sp.
Diptera	Calliphoridae	Bellardia pandia (Walker, 1849)
Diptera	Calliphoridae	Bellardia viarum (Robineau-Desvoidy, 1830)
Diptera	Calliphoridae	Bellardia vulgaris (Robineau-Desvoidy, 1830)
Diptera	Calliphoridae	Calliphoridae sp.
Diptera	Calliphoridae	Melinda viridicyanea (Robineau-Desvoidy, 1830)
Diptera	Calliphoridae	Pollenia amentaria (Scopoli, 1763)
Diptera	Calliphoridae	Pollenia atramentaria (Meigen, 1826)
Diptera	Calliphoridae	Pollenia hungarica Rognes, 1987
Diptera	Calliphoridae	Pollenia pediculata Macquart, 1834
Diptera	Calliphoridae	Pollenia sp.
Diptera	Cecidomyiidae	Cecidomyiidae sp.
Diptera	Conopidae	Myopa buccata (Linnaeus, 1758)
Diptera	Conopidae	Physocephala rufipes (Fabricius, 1781)
Diptera	Conopidae	Sicus ferrugineus (Linnaeus, 1761)
Diptera	Conopidae	Thecophora atra (Fabricius, 1775)
Diptera	Conopidae	Zodion cinereum (Fabricius 1794)
Diptera	Conopidae	Zodion nigitarsis (Strobl, 1902)
Diptera	Dolichopodidae	Dolichopodidae sp.
Diptera	Empididae	Bicellaria sp.M1
Diptera	Empididae	Coptophlebia leptomorion Bezzi, 1909
Diptera	Empididae	Drapetis sp.
Diptera	Empididae	Drapetis sp.M1
Diptera	Empididae	Empididae sp.
Diptera	Empididae	Empis caudatula Loew, 1867
Diptera	Empididae	Empis cf. florisomma
Diptera	Empididae	Empis cf. planetica
Diptera	Empididae	Empis cf. simulium
Diptera	Empididae	Empis fasciculata Strobl, 1901
Diptera	Empididae	Empis malleola Becker, 1887
Diptera	Empididae	Empis nigripes Fabricius, 1794
Diptera	Empididae	Empis nuntia Meigen, 1838
Diptera	Empididae	Empis sp.
Diptera	Empididae	Empis sp.M1
Diptera	Empididae	Empis sp.M2
Diptera	Empididae	Empis sp.M3
Diptera	Empididae	Empis sp.M4
Diptera	Empididae	Empis sp.M5
Diptera	Empididae	Euempis ciliata Fabricius, 1787

Diptera	Empididae	Euempis tessellata Fabricius, 1794
Diptera	Empididae	Hilara sp.M1
Diptera	Empididae	Hilara sp.M2
Diptera	Empididae	Hilara sp.M3
Diptera	Empididae	Holoclera flaviventris Macquart, 1827
Diptera	Empididae	Kritempis livida Linnaeus, 1758
Diptera	Empididae	Leptempis pandellei Daugeron, 1999
Diptera	Empididae	Leptempis rustica Fallén, 1816
Diptera	Empididae	Leptempis variegata Meigen, 1804
Diptera	Empididae	Lundstroemiella brevistylata Oldenberg, 1927
Diptera	Empididae	Lundstroemiella dudai Oldenberg, 1927
Diptera	Empididae	Lundstroemiella sp.M3
Diptera	Empididae	Pachymeria sp.
Diptera	Empididae	Pachymeria sp.M1
Diptera	Empididae	Pachymeria sp.M2
Diptera	Empididae	Pachymeria tumida Meigen, 1822
Diptera	Empididae	Rhamphomyia cf. Alpinomyia sp.M1
Diptera	Empididae	Rhamphomyia sp.M1
Diptera	Empididae	Rhamphomyia sp.M2
Diptera	Empididae	Rhamphomyia tibialis Meigen, 1822
Diptera	Empididae	Tachypeza sp.M1
Diptera	Empididae	Trichina sp.M1
Diptera	Empididae	Xanthempis pseudosemicinerea Daugeron, 2000
Diptera	Empididae	Xanthempis semicinerea Loew, 1867
Diptera	fam_indet	Diptera sp.
Diptera	Hybotidae	Hybotidae sp.
Diptera	Muscidae	Coenosia obscuricula (Rondani, 1871)
Diptera	Muscidae	Coenosia sp.
Diptera	Muscidae	Coenosia verralli Collin, 1953
Diptera	Muscidae	Drymeia alpicola (Rondani, 1871)
Diptera	Muscidae	Drymeia brumalis (Rondani, 1866)
Diptera	Muscidae	Drymeia fasciculata (Stein, 1916)
Diptera	Muscidae	Drymeia vicana (Harris, 1780)
Diptera	Muscidae	Helina latitarsis Ringdahl, 1924
Diptera	Muscidae	Helina reversio (Harris, 1780)
Diptera	Muscidae	Helina setiventris Ringdahl, 1924
Diptera	Muscidae	Helina sp.
Diptera	Muscidae	Hydrotaea irritans (Fallén, 1823)
Diptera	Muscidae	Musca domestica Linnaeus, 1758
Diptera	Muscidae	Musca osiris Wiedemann, 1830
Diptera	Muscidae	Muscidae sp.
Diptera	Muscidae	Mydaea rufinervis (Pokorny, 1889)
Diptera	Muscidae	Phaonia alpicola (Zetterstedt, 1845)
Diptera	Muscidae	Phaonia angelicae (Scopoli, 1763)

Diptera	Muscidae	Phaonia consobrina (Zetterstedt, 1838)
Diptera	Muscidae	Phaonia disjuncta Stein, 1916
Diptera	Muscidae	Phaonia incana (Wiedemann, 1817)
Diptera	Muscidae	Phaonia meigeni Pont, 1986
Diptera	Muscidae	Phaonia serva (Meigen, 1826)
Diptera	Muscidae	Spilogona dispar (Fallen, 1823)
Diptera	Muscidae	Thricops aculeipes (Zetterstedt, 1838)
Diptera	Muscidae	Thricops culminum (Pokorny, 1889)
Diptera	Muscidae	Thricops cunctans (Meigen, 1826)
Diptera	Muscidae	Thricops innocuus (Zetterstedt, 1838)
Diptera	Muscidae	Thricops longipes (Zetterstedt, 1845)
Diptera	Muscidae	Thricops nigrifrons (Robineau-Desvoidy, 1830)
Diptera	Muscidae	Thricops nigritellus (Zetterstedt, 1838)
Diptera	Muscidae	Thricops rostratus (Meade, 1882)
Diptera	Muscidae	Thricops semicinereus (Wiedemann, 1817)
Diptera	Mycetophilidae	Mycetophilidae sp.
Diptera	Phoridae	Phoridae sp.
Diptera	Rhinophoridae	Rhinophoridae sp.
Diptera	Sarcophagidae	Sarcophagidae sp.
Diptera	Sciomyzidae	Sciomyzidae sp.
Diptera	Stratiomyidae	Chloromyia formosa (Scopoli), 1763
Diptera	Stratiomyidae	Nemotelus nigrinus Fallen, 1817
Diptera	Stratiomyidae	Nemotelus pantherinus (Linnaeus, 1758)
Diptera	Stratiomyidae	Sargus flavipes Meigen, 1822
Diptera	Syrphidae	Brachypalpoides lentus (Meigen, 1822)
Diptera	Syrphidae	Chamaesyrphus scaevoides (Fallen, 1817)
Diptera	Syrphidae	Cheilosia albitarsis (Meigen, 1822)
Diptera	Syrphidae	Cheilosia albitarsis agg.
Diptera	Syrphidae	Cheilosia antiqua (Meigen, 1822)
Diptera	Syrphidae	Cheilosia barbata Loew, 1857
Diptera	Syrphidae	Cheilosia bracusi Vujic & Claussen, 1994
Diptera	Syrphidae	Cheilosia caerulescens (Meigen, 1822)
Diptera	Syrphidae	Cheilosia gigantea (Zetterstedt, 1838)
Diptera	Syrphidae	Cheilosia himantopus (Panzer, 1798)
Diptera	Syrphidae	Cheilosia hypena (Becker, 1894)
Diptera	Syrphidae	Cheilosia impudens (Becker, 1894)
Diptera	Syrphidae	Cheilosia latifrons (Zetterstedt, 1843)
Diptera	Syrphidae	Cheilosia melanura (Becker, 1894)
Diptera	Syrphidae	Cheilosia mutabilis (Fallen, 1817)
Diptera	Syrphidae	Cheilosia nebulosa (Verrall, 1871)
Diptera	Syrphidae	Cheilosia proxima (Zetterstedt, 1843)
Diptera	Syrphidae	Cheilosia ranunculi Doczkal, 2000
Diptera	Syrphidae	Cheilosia semifasciata (Becker, 1894)
Diptera	Syrphidae	Cheilosia sp.

Diptera	Syrphidae	Cheilosia urbana (Meigen, 1822)
Diptera	Syrphidae	Cheilosia vangaveri (Timon-David, 1937)
Diptera	Syrphidae	Cheilosia variabilis (Panzer, 1798)
Diptera	Syrphidae	Cheilosia velutina Loew, 1840
Diptera	Syrphidae	Cheilosia vernalis (Fallen, 1817)
Diptera	Syrphidae	Cheilosia vicina (Zetterstedt, 1849)
Diptera	Syrphidae	Cheilosia vulpina (Meigen, 1822)
Diptera	Syrphidae	Chrysotoxum arcuatum (Linnaeus, 1758)
Diptera	Syrphidae	Chrysotoxum bicinctum (Linnaeus, 1758)
Diptera	Syrphidae	Chrysotoxum elegans Loew, 1841
Diptera	Syrphidae	Chrysotoxum festivum (Linnaeus, 1758)
Diptera	Syrphidae	Chrysotoxum octomaculatum Curtis, 1837
Diptera	Syrphidae	Chrysotoxum vernale Loew, 1841
Diptera	Syrphidae	Chrysotoxum verralli Collin, 1940
Diptera	Syrphidae	Dasysyrphus friuliensis (van der Goot, 1960)
Diptera	Syrphidae	Dasysyrphus pinastri (De Geer, 1776)
Diptera	Syrphidae	Dasysyrphus postclaviger (Stys & Moucha, 1962)
Diptera	Syrphidae	Dasysyrphus tricinctus (Fallen, 1817)
Diptera	Syrphidae	Dasysyrphus venustus (Meigen, 1822)
Diptera	Syrphidae	Episyrphus balteatus Geer, 1776
Diptera	Syrphidae	Eristalis arbustorum (Linnaeus, 1758)
Diptera	Syrphidae	Eristalis jugorum Egger, 1858
Diptera	Syrphidae	Eristalis tenax Linnaeus, 1758
Diptera	Syrphidae	Eupeodes corollae Fabricius, 1794
Diptera	Syrphidae	Eupeodes latifasciatus (Macquart, 1829)
Diptera	Syrphidae	Eupeodes luniger (Meigen, 1822)
Diptera	Syrphidae	Eupeodes nitens (Zetterstedt, 1843)
Diptera	Syrphidae	Lapposyrphus lapponicus (Zetterstedt, 1838)
Diptera	Syrphidae	Melanogaster nuda (Macquart, 1829)
Diptera	Syrphidae	Melanostoma mellinum (Linnaeus, 1758)
Diptera	Syrphidae	Merodon armipes Rondani, 1843
Diptera	Syrphidae	Merodon aureus Fabricius, 1805
Diptera	Syrphidae	Merodon cinereus (Fabricius, 1794)
Diptera	Syrphidae	Merodon equestris (Fabricius, 1794)
Diptera	Syrphidae	Neocnemodon vitripennis (Meigen, 1822)
Diptera	Syrphidae	Orthonevra nobilis (Fallen, 1817)
Diptera	Syrphidae	Paragus bicolor (Fabricius, 1794)
Diptera	Syrphidae	Paragus finitimus Goeldlin, 1971
Diptera	Syrphidae	Parasyrphus annulatus (Zetterstedt, 1838)
Diptera	Syrphidae	Parasyrphus lineolus (Zetterstedt, 1843)
Diptera	Syrphidae	Parasyrphus punctulatus (Verrall, 1873)
Diptera	Syrphidae	Parasyrphus vittiger (Zetterstedt, 1843)
Diptera	Syrphidae	Pipiza noctiluca (Linnaeus, 1758)
Diptera	Syrphidae	Pipiza quadrimaculata (Panzer, 1804)

Diptera	Syrphidae	Pipizella annulata Macquart, 1829
Diptera	Syrphidae	Pipizella calabra (Goeldlin, 1974)
Diptera	Syrphidae	Pipizella sp.
Diptera	Syrphidae	Pipizella viduata (Linnaeus, 1758)
Diptera	Syrphidae	Pipizella zeneggenensis (Goeldlin, 1974)
Diptera	Syrphidae	Platycheirus albimanus (Fabricius, 1781)
Diptera	Syrphidae	Platycheirus cf. ambiguus (Fallen, 1817)
Diptera	Syrphidae	Platycheirus manicatus (Meigen, 1822)
Diptera	Syrphidae	Platycheirus tarsalis (Schummel, 1836)
Diptera	Syrphidae	Platycheirus tatricus Dusek & Laska, 1982
Diptera	Syrphidae	Portevinia maculata (Fallen, 1817)
Diptera	Syrphidae	Rhingia campestris Meigen, 1822
Diptera	Syrphidae	Rhingia rostrata (Linnaeus, 1758)
Diptera	Syrphidae	Scaeva pyrastri (Linnaeus, 1758)
Diptera	Syrphidae	Scaeva selenitica (Meigen, 1822)
Diptera	Syrphidae	Sphaerophoria interrupta Jones, 1917
Diptera	Syrphidae	Sphaerophoria interrupta agg. (Fabricius, 1805)
Diptera	Syrphidae	Sphaerophoria laurae Goeldlin, 1989
Diptera	Syrphidae	Sphaerophoria scripta (Linnaeus, 1758)
Diptera	Syrphidae	Sphaerophoria sp.
Diptera	Syrphidae	Sphaerophoria taeniata (Meigen, 1822)
Diptera	Syrphidae	Sphaeroria sp.
Diptera	Syrphidae	Syritta pipiens (Linnaeus, 1758)
Diptera	Syrphidae	Syrphidae sp.
Diptera	Syrphidae	Syrphus ribesii (Linnaeus, 1758)
Diptera	Syrphidae	Syrphus torvus Osten Sacken, 1875
Diptera	Syrphidae	Syrphus vitripennis Meigen, 1822
Diptera	Syrphidae	Volucella bombylans (Linnaeus, 1758)
Diptera	Syrphidae	Volucella pellucens (Linnaeus, 1758)
Diptera	Tachinidae	Acemya acuticornis (Meigen, 1824)
Diptera	Tachinidae	Besseria anthophila (Loew, 1871)
Diptera	Tachinidae	Clytiomya sola (Rondani, 1861)
Diptera	Tachinidae	Cylindromyia brassicaria (Fabricius, 1775)
Diptera	Tachinidae	Dinera carinifrons (Fallen, 1817)
Diptera	Tachinidae	Gymnosoma dolycoridis Dupuis, 1961
Diptera	Tachinidae	Gymnosoma nitens Meigen, 1824
Diptera	Tachinidae	Gymnosoma nudifrons Herting, 1966
Diptera	Tachinidae	Minthodes picta (Zetterstedt, 1844)
Diptera	Tachinidae	Peleteria ferina (Zetterstedt, 1844)
Diptera	Tachinidae	Phania funesta (Meigen, 1824)
Diptera	Tachinidae	Phasia obesa (Fabricius, 1798)
Diptera	Tachinidae	Phasia pusilla Meigen, 1824
Diptera	Tachinidae	Siphona flavifrons Stæger, 1849
Diptera	Tachinidae	Siphona geniculata (De Geer, 1776)

Diptera	Tachinidae	Siphona maculata Staeger, 1849
Diptera	Tachinidae	Tachina fera Linnaeus, 1761
Diptera	Tachinidae	Tachinidae sp.
Diptera	Tachinidae	Zophomyia temula (Scopoli, 1763)
Diptera	Tephritidae	Orellia falcata (Scopoli, 1763)
Heteroptera	Coreidae	Coriomeris affinis (Herrich-Schäffer, 1839)
Heteroptera	Pentatomidae	Peribalus strictus vernalis (Wolff, 1804)
Hymenoptera	Andrenidae	Andrena alfkenella Perkins, 1914
Hymenoptera	Andrenidae	Andrena combinata (Christ, 1791)
Hymenoptera	Andrenidae	Andrena fulvago (Christ, 1791)
Hymenoptera	Andrenidae	Andrena gelriae van der Vecht, 1927
Hymenoptera	Andrenidae	Andrena hattorfiana (Fabricius, 1775)
Hymenoptera	Andrenidae	Andrena intermedia Thomson, 1870
Hymenoptera	Andrenidae	Andrena labialis (Kirby, 1802)
Hymenoptera	Andrenidae	Andrena labiata Fabricius, 1781
Hymenoptera	Andrenidae	Andrena minutuloides Perkins, 1914
Hymenoptera	Andrenidae	Andrena montana Warncke, 1973
Hymenoptera	Andrenidae	Andrena nuptialis Pérez, 1902
Hymenoptera	Andrenidae	Andrena paucisquama Noskiewicz, 1924
Hymenoptera	Andrenidae	Andrena saxonica Stoeckhert, 1935
Hymenoptera	Andrenidae	Andrena similis Smith, 1849
Hymenoptera	Andrenidae	Andrena subopaca Nylander, 1848
Hymenoptera	Andrenidae	Andrena tarsata Nylander, 1848
Hymenoptera	Andrenidae	Andrena wilkella (Kirby, 1802)
Hymenoptera	Andrenidae	Panurginus montanus Giraud, 1861
Hymenoptera	Andrenidae	Panurgus banksianus (Kirby, 1802)
Hymenoptera	Apidae	Apis mellifera Linnaeus, 1758
Hymenoptera	Apidae	Bombus gr. terrestris (Linnaeus, 1758)
Hymenoptera	Apidae	Bombus hortorum Linnaeus, 1761
Hymenoptera	Apidae	Bombus humilis Illiger, 1806
Hymenoptera	Apidae	Bombus lapidarius Linnaeus, 1758
Hymenoptera	Apidae	Bombus lucorum (Linnaeus, 1761)
Hymenoptera	Apidae	Bombus mendax Gerstäcker, 1869
Hymenoptera	Apidae	Bombus mucidus Gerstäcker, 1869
Hymenoptera	Apidae	Bombus pratorum Illiger, 1806
Hymenoptera	Apidae	Bombus ruderarius Müller, 1776
Hymenoptera	Apidae	Bombus sichelii Radoszkowski, 1860
Hymenoptera	Apidae	Bombus soroeensis Fabricius, 1776
Hymenoptera	Apidae	Bombus sp.
Hymenoptera	Apidae	Bombus wurflenii Radoszkowski, 1860
Hymenoptera	Apidae	Ceratina chalybea Chevrier, 1872
Hymenoptera	Apidae	Ceratina cyanea (Kirby, 1802)
Hymenoptera	Apidae	Eucera longicornis (Linnaeus, 1758)
Hymenoptera	Apidae	Nomada distinguenda Morawitz, 1874

Hymenoptera	Apidae	Nomada flavoguttata (Kirby, 1802)
Hymenoptera	Apidae	Nomada goodeniana (Kirby, 1802)
Hymenoptera	Argidae	Arge cyanocrocea (Forster, 1771)
Hymenoptera	Argidae	Arge nigripes (Retzius, 1783)
Hymenoptera	Cephidae	Cephus pygmeus (Linnaeus, 1767)
Hymenoptera	Chrysididae	Chrysididae sp.1
Hymenoptera	Cimbicidae	Corynis crassicornis (Rossi, 1790)
Hymenoptera	Cimbicidae	Corynis obscura (Fabricius, 1775)
Hymenoptera	Colletidae	Colletes similis Schenck, 1853
Hymenoptera	Colletidae	Hylaeus angustatus (Schenck, 1861)
Hymenoptera	Colletidae	Hylaeus annularis (Kirby, 1802)
Hymenoptera	Colletidae	Hylaeus cf. annulatus (Linnaeus, 1758)
Hymenoptera	Colletidae	Hylaeus confusus Nylander, 1852
Hymenoptera	Colletidae	Hylaeus gredleri Förster, 1871
Hymenoptera	Crabronidae	Bembix sp.
Hymenoptera	Crabronidae	Crabro sp.
Hymenoptera	Halictidae	Dufourea alpina Morawitz, 1865
Hymenoptera	Halictidae	Dufourea dentiventris (Nylander, 1848)
Hymenoptera	Halictidae	Halictus gr. simplex Blüthgen, 1923
Hymenoptera	Halictidae	Halictus maculatus Smith, 1848
Hymenoptera	Halictidae	Halictus quadricinctus (Fabricius, 1776)
Hymenoptera	Halictidae	Halictus rubicundus (Christ, 1791)
Hymenoptera	Halictidae	Halictus scabiosae (Rossi, 1790)
Hymenoptera	Halictidae	Halictus smaragdulus s.l. Vachal, 1895
Hymenoptera	Halictidae	Halictus subauratus (Rossi, 1792)
Hymenoptera	Halictidae	Halictus tumulorum (Linnaeus, 1758)
Hymenoptera	Halictidae	Lasioglossum aeratum (Kirby, 1802)
Hymenoptera	Halictidae	Lasioglossum albipes (Fabricius, 1781)
Hymenoptera	Halictidae	Lasioglossum alpigenum (Dalla Torre, 1877)
Hymenoptera	Halictidae	Lasioglossum brevicorne (Schenck, 1870)
Hymenoptera	Halictidae	Lasioglossum calceatum (Scopoli, 1763)
Hymenoptera	Halictidae	Lasioglossum fratellum s.l. (Pérez, 1903)
Hymenoptera	Halictidae	Lasioglossum fulvicorne (Kirby, 1802)
Hymenoptera	Halictidae	Lasioglossum gr. pauxillum (Schenck, 1853)
Hymenoptera	Halictidae	Lasioglossum interruptum (Panzer, 1798)
Hymenoptera	Halictidae	Lasioglossum laeve (Kirby, 1802)
Hymenoptera	Halictidae	Lasioglossum laevigatum (Kirby, 1802)
Hymenoptera	Halictidae	Lasioglossum laticeps (Schenck, 1870)
Hymenoptera	Halictidae	Lasioglossum lativentre (Schenck, 1853)
Hymenoptera	Halictidae	Lasioglossum leucopus (Kirby, 1802)
Hymenoptera	Halictidae	Lasioglossum leucozonium (Schrank, 1781)
Hymenoptera	Halictidae	Lasioglossum minutulum (Schenck, 1853)
Hymenoptera	Halictidae	Lasioglossum morio (Fabricius, 1793)
Hymenoptera	Halictidae	Lasioglossum nigripes (Lepeletier, 1841)

Hymenoptera	Halictidae	Lasioglossum pauxillum (Schenck, 1853)
Hymenoptera	Halictidae	Lasioglossum politum (Schenck, 1853)
Hymenoptera	Halictidae	Lasioglossum sp.1
Hymenoptera	Halictidae	Lasioglossum sp.2
Hymenoptera	Halictidae	Lasioglossum tricinctum (Schenck, 1874)
Hymenoptera	Halictidae	Lasioglossum villosulum (Kirby, 1802)
Hymenoptera	Halictidae	Lasioglossum xanthopus (Kirby, 1802)
Hymenoptera	Halictidae	Lasioglossum zonulum (Smith, 1848)
Hymenoptera	Halictidae	Sphecodes ephippius (Linnaeus, 1767)
Hymenoptera	Halictidae	Sphecodes reticulatus Thomson, 1870
Hymenoptera	Ichneumonidae	Banchinae sp.1
Hymenoptera	Ichneumonidae	Banchinae sp.2
Hymenoptera	Ichneumonidae	Banchinae sp.3
Hymenoptera	Ichneumonidae	Campopleginae sp.1
Hymenoptera	Ichneumonidae	Campopleginae sp.2
Hymenoptera	Ichneumonidae	Ichneumoninae sp.1
Hymenoptera	Ichneumonidae	Ichneumoninae sp.2
Hymenoptera	Ichneumonidae	Ichneumoninae sp.3
Hymenoptera	Megachilidae	Chelostoma distinctum (Stoeckhert, 1929)
Hymenoptera	Megachilidae	Chelostoma florisomne Linnaeus, 1758
Hymenoptera	Megachilidae	Chelostoma grande Linnaeus, 1758
Hymenoptera	Megachilidae	Heriades truncorum (Linnaeus, 1758)
Hymenoptera	Megachilidae	Megachile analis Nylander, 1852
Hymenoptera	Megachilidae	Megachile circumcincta (Kirby, 1802)
Hymenoptera	Megachilidae	Megachile pyrenaea Pérez, 1890
Hymenoptera	Megachilidae	Megachile sp.
Hymenoptera	Megachilidae	Megachile willughbiella (Kirby, 1802)
Hymenoptera	Megachilidae	Osmia bicornis Linnaeus, 1758
Hymenoptera	Megachilidae	Osmia cerinthidis Morawitz, 1876
Hymenoptera	Megachilidae	Osmia labialis Pérez, 1879
Hymenoptera	Megachilidae	Osmia leaiana (Kirby, 1802)
Hymenoptera	Megachilidae	Osmia scutellaris Morawitz, 1868
Hymenoptera	Megachilidae	Osmia sp.
Hymenoptera	Megachilidae	Osmia spinulosa (Kirby, 1802)
Hymenoptera	Megachilidae	Stelis franconica Blüthgen, 1930
Hymenoptera	Megalodontesidae	Megalodontes cephalotes (Fabricius, 1781)
Hymenoptera	Megalodontesidae	Megalodontes plagiocephalus (Fabricius, 1804)
Hymenoptera	Tenthredinidae	Aglaostigma aucupariae (Klug, 1817)
Hymenoptera	Tenthredinidae	Athalia bicolor Serville, 1823
Hymenoptera	Tenthredinidae	Empria sp.
Hymenoptera	Tenthredinidae	Monophadnus alpicola Benson, 1954
Hymenoptera	Tenthredinidae	Tenthredo arcuata Forster, 1771
Hymenoptera	Tenthredinidae	Tenthredo bifasciata O. F. Muller, 1766
Hymenoptera	Tenthredinidae	Tenthredo brevicornis (Konow, 1886)

Hymenoptera	Tenthredinidae	Tenthredo frauenfeldii Giraud, 1857
Hymenoptera	Tenthredinidae	Tenthredo koehleri Klug, 1817
Hymenoptera	Tenthredinidae	Tenthredo mesomela Linnaeus, 1758
Hymenoptera	Tenthredinidae	Tenthredo schaefferi Klug, 1817
Hymenoptera	Tenthredinidae	Tenthredo sp.
Hymenoptera	Tenthredinidae	Tenthredo velox Fabricius, 1798
Hymenoptera	Vespidae	Eumeninae sp.1
Hymenoptera	Vespidae	Polistes sp.
Lepidoptera	Adelidae	Adelidae sp.
Lepidoptera	fam_indet	Lepidoptera sp.
Lepidoptera	Hesperiidae	Hesperiidae sp.
Lepidoptera	Hesperiidae	Ochlodes sp.
Lepidoptera	Hesperiidae	Thymelicus sp.
Lepidoptera	Hesperiidae	Thymelicus sylvestris (Poda, 1761)
Lepidoptera	Lycaenidae	Lycaenidae sp.
Lepidoptera	Lycaenidae	Polyommatus icarus (Rottemburg, 1775)
Lepidoptera	Nymphalidae	Aglais io (Linnaeus, 1758)
Lepidoptera	Nymphalidae	Coenonympha sp.
Lepidoptera	Nymphalidae	Erebia alberganus (Prunner, 1798)
Lepidoptera	Nymphalidae	Erebia sp.
Lepidoptera	Nymphalidae	Maniola jurtina (Linnaeus, 1758)
Lepidoptera	Nymphalidae	Melanargia galathea (Linnaeus, 1758)
Lepidoptera	Nymphalidae	Melitaea parthenoides Keferstein, 1851
Lepidoptera	Nymphalidae	Melitaea sp.
Lepidoptera	Nymphalidae	Nymphalidae sp.
Lepidoptera	Nymphalidae	Satyrium sp.
Lepidoptera	Papilionidae	Iphiclides podalirius (Linnaeus, 1758)
Lepidoptera	Pieridae	Anthocharis cardamines Linnaeus, 1758
Lepidoptera	Pieridae	Aporia crataegi Linnaeus, 1758
Lepidoptera	Pieridae	Pieridae sp.
Lepidoptera	Pieridae	Pieris sp.
Lepidoptera	Sphingidae	Hemaris fuciformis (Linnaeus, 1758)
Lepidoptera	Sphingidae	Macroglossum stellatarum (Linnaeus, 1758)
Lepidoptera	Zygaenidae	Adscita sp.
Lepidoptera	Zygaenidae	Zygaena sp.
Lepidoptera	Zygaenidae	Zygaena viciae Denis & Schiffermüller, 1775
Lepidoptera	Zygaenidae	Zygaenidae sp.
Neuroptera	Chrysopidae	Chrysopidae sp.

Figure S11 : Species-level flower visitation networks illustrating the overlap in resource use between each pair of the three main anthophilous orders: Diptera (blue), Hymenoptera (yellow), Coleoptera (green). Flower codes : see Table S15.

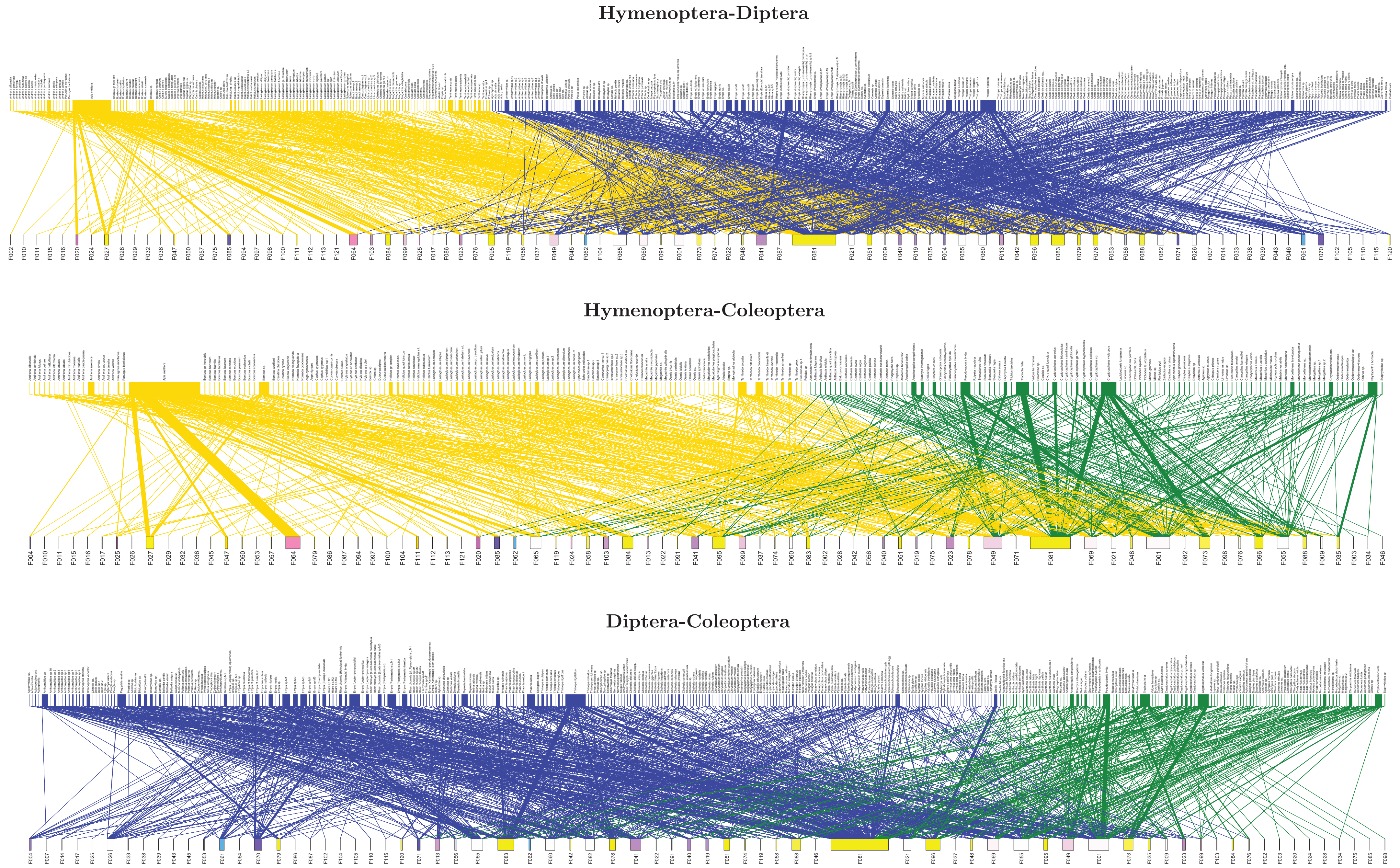

Figure S12 (1/2): Species-level flower visitation networks illustrating the overlap in resource use between each pair of the four main anthophilous fly families: Anthomyiidae (purple), Empididae (blue), Muscidae (green), Syrphidae (yellow). Flower codes : see Table S15.

Syrphidae-Empididae

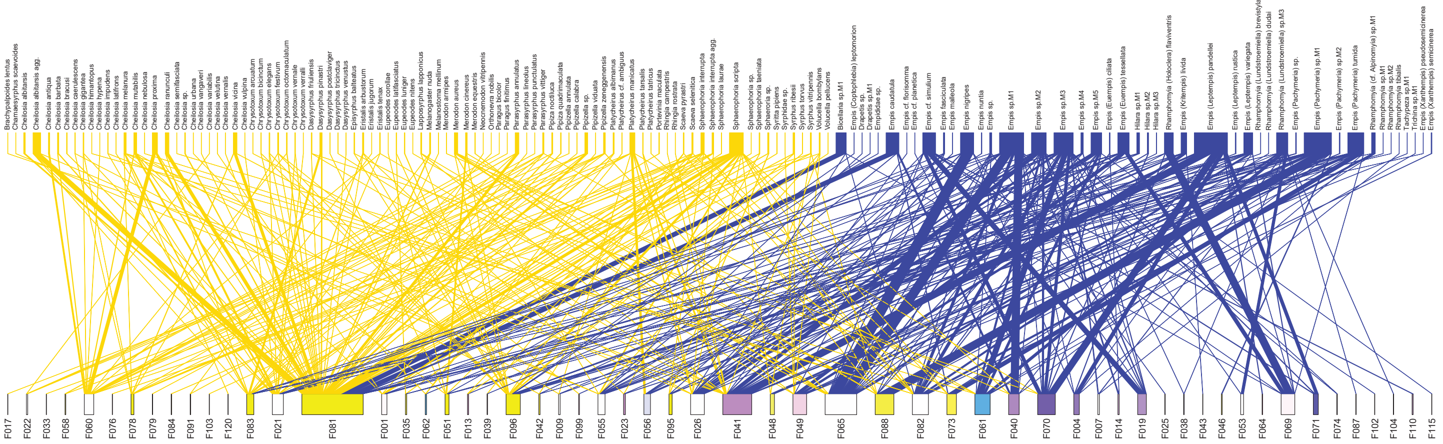

Syrphidae-Anthomyiidae

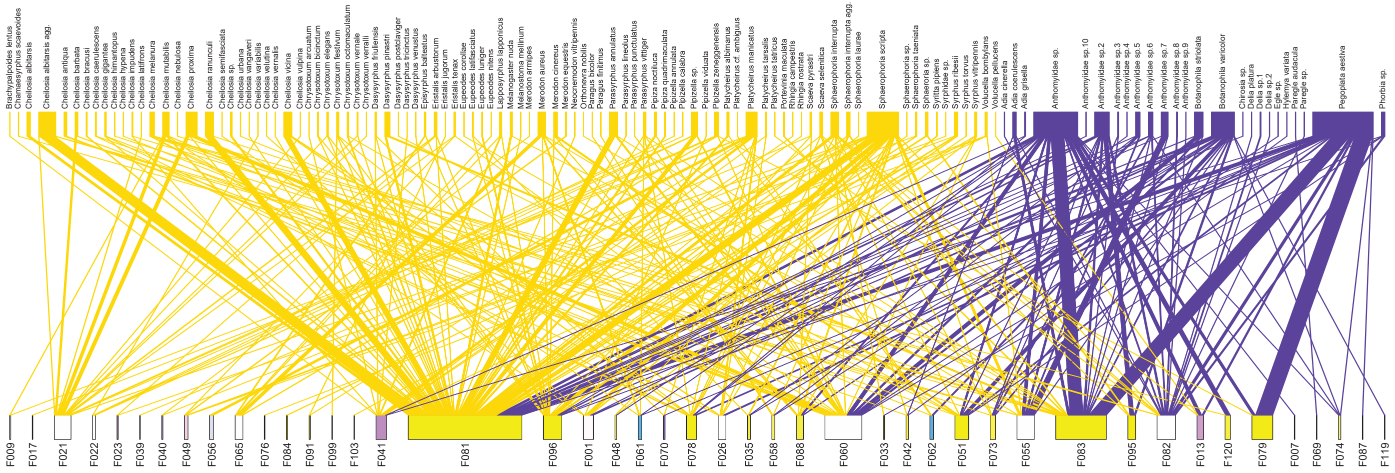

Syrphidae-Muscidae

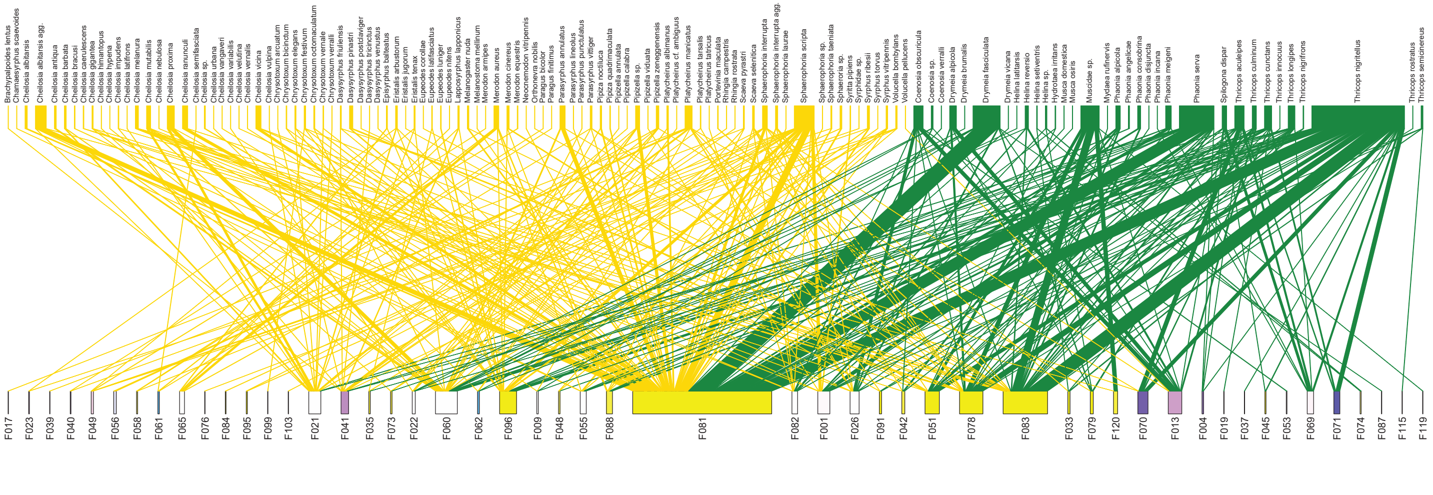

Figure S12 (2/2)

Empididae-Anthomyiidae

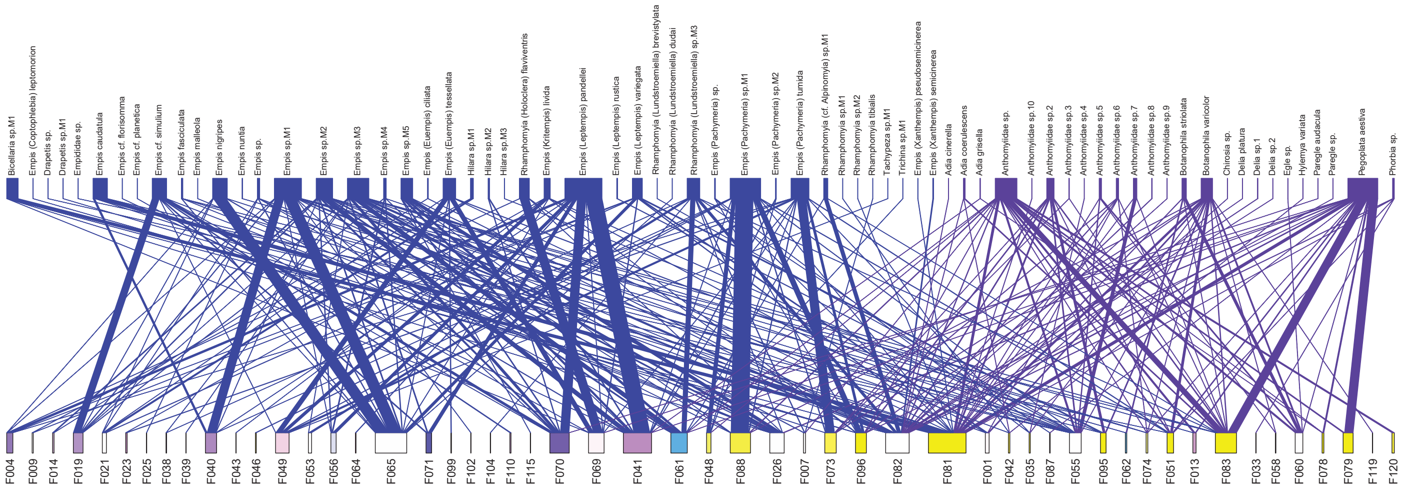

Empididae-Muscidae

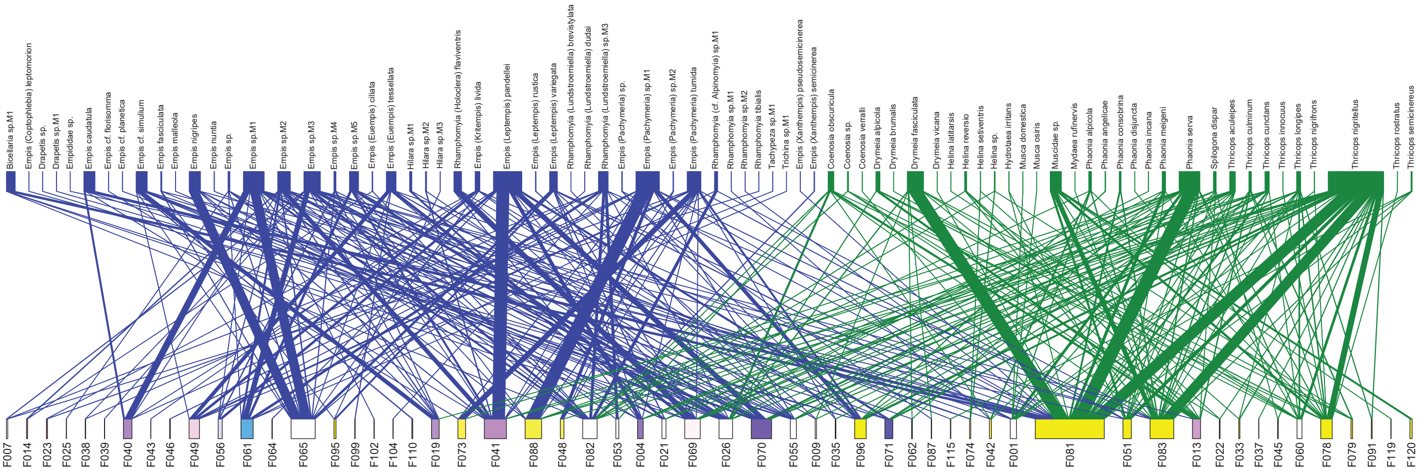

Muscidae-Anthomyiidae

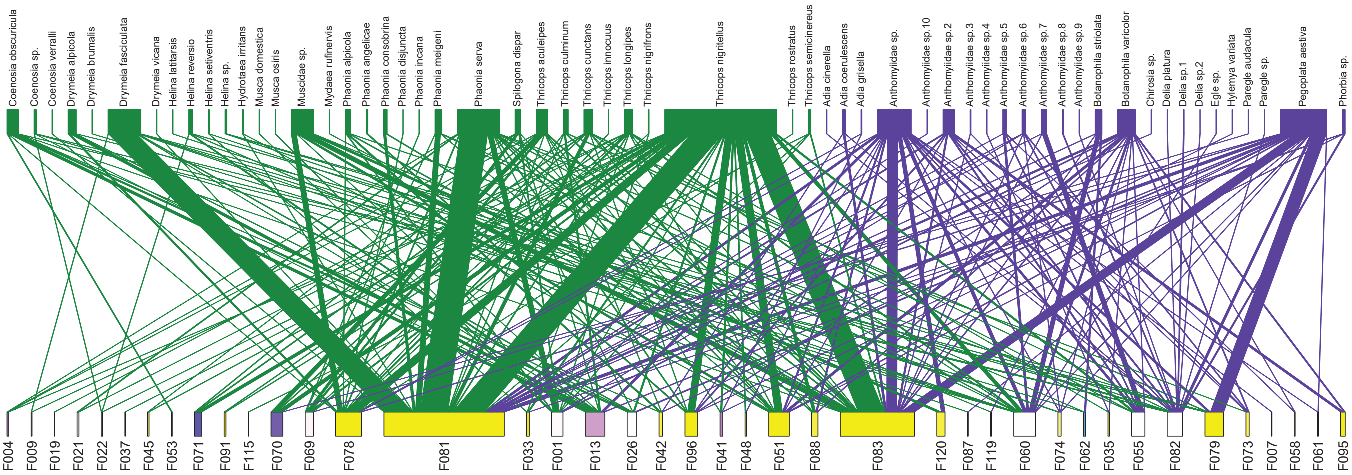

Supplement: Supplementary file 1 — Supplementary material [file 41598_2018_23210_MOESM1_ESM.pdf]
